# Copper chelation suppresses epithelial-mesenchymal transition by inhibition of canonical and non-canonical TGF-β signaling pathways in cancer

**DOI:** 10.1186/s13578-023-01083-7

**Published:** 2023-07-21

**Authors:** Ensieh M. Poursani, Daniele Mercatelli, Prahlad Raninga, Jessica L. Bell, Federica Saletta, Felix V. Kohane, Daniel P. Neumann, Ye Zheng, Jourdin R. C. Rouaen, Toni Rose Jue, Filip T. Michniewicz, Piper Schadel, Erin Kasiou, Maria Tsoli, Giuseppe Cirillo, Shafagh Waters, Tyler Shai-Hee, Riccardo Cazzoli, Merryn Brettle, Iveta Slapetova, Maria Kasherman, Renee Whan, Fernando Souza-Fonseca-Guimaraes, Linda Vahdat, David Ziegler, John G. Lock, Federico M. Giorgi, KumKum Khanna, Orazio Vittorio

**Affiliations:** 1 Children’s Cancer Institute Australia, Lowy Cancer Research Centre, University of New South Wales, Sydney, NSW Australia; 2 School of Biomedical Sciences, Faculty of Medicine and Health, University of New South Wales, Sydney, NSW Australia; 3 Department of Pharmacy and Biotechnology, University of Bologna, Bologna, Italy; 4 QIMR Berghofer Medical Research Institute, Brisbane, QLD Australia; 5 Department of Pharmacy, Health and Nutritional Sciences, University of Calabria, Rende, Italy; 6 Department of Experimental Oncology, IEO, European Institute of Oncology IRCCS, Milan, Italy; 7 Katharina Gauss Light Microscopy Facility, University of New South Wales, Sydney, NSW Australia; 8 Dartmouth Cancer Center, Lebanon, NH USA; 9 Kids Cancer Centre, Sydney Children’s Hospital, Randwick, NSW Australia; 10 Garvan Institute of Medical Research, Darlinghurst, NSW Australia; 11 The University of Queensland Diamantina Institute, The University of Queensland, Woolloongabba, QLD Australia

**Keywords:** Cancer, Copper chelation, EMT, Metastasis, TGF-β signaling pathways

## Abstract

**Background:**

Metastatic cancer cells exploit Epithelial-mesenchymal-transition (EMT) to enhance their migration, invasion, and resistance to treatments. Recent studies highlight that elevated levels of copper are implicated in cancer progression and metastasis. Clinical trials using copper chelators are associated with improved patient survival; however, the molecular mechanisms by which copper depletion inhibits tumor progression and metastasis are poorly understood. This remains a major hurdle to the clinical translation of copper chelators. Here, we propose that copper chelation inhibits metastasis by reducing TGF-β levels and EMT signaling. Given that many drugs targeting TGF-β have failed in clinical trials, partly because of severe side effects arising in patients, we hypothesized that copper chelation therapy might be a less toxic alternative to target the TGF-β/EMT axis.

**Results:**

Our cytokine array and RNA-seq data suggested a link between copper homeostasis, TGF-β and EMT process. To validate this hypothesis, we performed single-cell imaging, protein assays, and in vivo studies. Here, we used the copper chelating agent TEPA to block copper trafficking. Our in vivo study showed a reduction of TGF-β levels and metastasis to the lung in the TNBC mouse model. Mechanistically, TEPA significantly downregulated canonical (TGF-β/SMAD2&3) and non-canonical (TGF-β/PI3K/AKT, TGF-β/RAS/RAF/MEK/ERK, and TGF-β/WNT/β-catenin) TGF-β signaling pathways. Additionally, EMT markers of MMP-9, MMP-14, Vimentin, β-catenin, ZEB1, and p-SMAD2 were downregulated, and EMT transcription factors of SNAI1, ZEB1, and p-SMAD2 accumulated in the cytoplasm after treatment.

**Conclusions:**

Our study suggests that copper chelation therapy represents a potentially effective therapeutic approach for targeting TGF-β and inhibiting EMT in a diverse range of cancers.

**Supplementary Information:**

The online version contains supplementary material available at 10.1186/s13578-023-01083-7.

## Background

Epithelial-mesenchymal-transition (EMT), is an embryonic phenotypic plasticity program that is reactivated in cancer cells in a dynamic fashion to acquire features that confer invasiveness, dissemination, and chemo/immunotherapy resistance [1, 2]. EMT is the first critical step in developing metastasis and invasion in most types of human malignancies [3–5]. A number of important signaling pathways such as TGF-β, WNT, Notch and Hedgehog are involved in modulating EMT via several transcription factors including the SMADS, Twist, Snail and Slug families [6–8]. TGF-β is known as a major regulator of the EMT process [9, 10]. The canonical TGF-β signaling pathway acts through TGF-β binding to its receptor, resulting in the receptor-regulated SMADs(R-SMADs), SMAD2 and SMAD3, phosphorylation and subsequent formation of a trimer with SMAD4. This complex translocate to the nucleus, where it regulates the transcription of target genes [10, 11]. The non-canonical TGF-β signaling pathway stimulates phosphatidylinositol 3- kinase (PI3K) and its downstream pathways by phosphorylating AKT and promoting EMT signaling [14]. Due to its strong influence on EMT signaling through these pathways, TGF-β signaling has a central role in metastatic disease and has been subject to both molecular and clinical investigations as a target in many cancer types [12].

Different strategies such as ligand traps, antisense oligonucleotides (ASOs), small molecule receptor kinase inhibitors, and peptide aptamers have been successfully used to target and modulate TGF-β signaling in cancers in vitro, however these strategies have faced severe problems in clinical trial studies [9, 13–15]. Specifically, the drugs targeting TGF-β have failed in clinical trials, largely due to the severe side effects arising in patients [13–17].

In the search for novel strategies, investigations have highlighted the interactions between TGF-β and matrix metalloproteinases (MMPs). TGF-β has been demonstrated to activate several MMPs, including MMP-9 and MMP-14 [4, 5]. Likewise, MMP-2, MMP-9, and MMP-14 can induce the biological activity of TGF-β via cleavage of latent TGF-β-binding protein-1 [18, 19]. Furthermore, a positive feedback loop between TGF-β and MMP-9 which is mediated by the PI3K signaling pathway has been recently demonstrated, highlighting the complexity and strong integration of TGF-β with MMPs [20].

The interactions between TGF-β with MMPs are central to metastatic processes, as it has been reported that TGF-β-induced cell migration is mediated by MMP-14, which also regulates MMP-9 activation [21, 22]. TGF-β and MMP-9 are associated with migration and invasion, promoting metastasis in several malignancies [23]. Moreover, there is an interconnection between TGF-β activation and MMP-9 expression, as an overexpression of MMP-9 increases the malignancy of breast cancer cell lines, largely via the activation of the SMADs signaling pathway [24]. MMP-9 has been recognized as an essential factor in mediating drug and radio resistance and metastasis in NB patients [25]. Moreover, TGF-β plays an important role in influencing the tumor microenvironment [26–28].

Copper is an essential catalytic cofactor involved in many biological processes in the human body [29, 30]. Previous studies demonstrated that copper homeostasis is dysregulated in metastatic cancers [31]. Copper accumulation has been observed in several malignancies such as breast, colon, gastric and lung cancer [32]. Copper is also known to have high expression in the brain, with radioactive Cu_64_ used to image brain cancers and indicating copper dependency [33–36].

Copper chelation therapy was developed to treat Wilson’s disease, a genetic disorder which results in the excess accumulation of copper [37]. Copper chelators are approved for use in both adults and children and have recently attracted the attention of oncologists because of their proven ability to suppress tumor growth and angiogenesis in many pre-clinical studies [38, 39]. We previously reported that copper chelation deregulated phosphorylation of STAT3 and AKT which are important to the tumor and its microenvironment including blocking the PD-1/PD-L1 axis in neuroblastoma [40, 41]. Moreover, a recent study revealed that copper chelators inhibit tumor metastasis by collagen remodeling in the pre-metastatic niche in triple-negative breast cancer (TNBC) [30]. It should also be noted that TGF-β signaling is central in the deposition of collagen. However, the exact mechanism by which copper depletion reduced tumor metastasis in this model is still poorly understood, with very few transcriptomic datasets publicly available.

Due to the effects of copper chelators on AKT signaling which is involved in non-canonical TGF-β signaling, their documented effects on tumor microenvironment remodeling and their inhibition of metastasis through collagen remodeling, which often is a result of TGF-β signaling, we hypothesized that copper chelators may be able to inhibit TGF-β signaling.

In this study, we show for the first time that the copper chelator Tetraethylenepentamine (TEPA) significantly reduces TGF-β levels and subsequently inhibits both canonical and non-canonical TGF-β signaling pathways. As a demonstration of this, copper depletion also downregulated proteins involved in EMT-associated cancer invasion and metastasis in TNBC, NB and DIPG cell lines, and reduced metastasis in vivo*.* Our data support the potential repurposing of copper chelating agents as an effective therapeutic strategy for targeting TGF-β in metastatic and invasive tumors.

## Results

### Copper depletion inhibits the in vivo tumor growth in 4T1.2 syngeneic murine model of triple-negative breast cancer

**Fig. 1:**
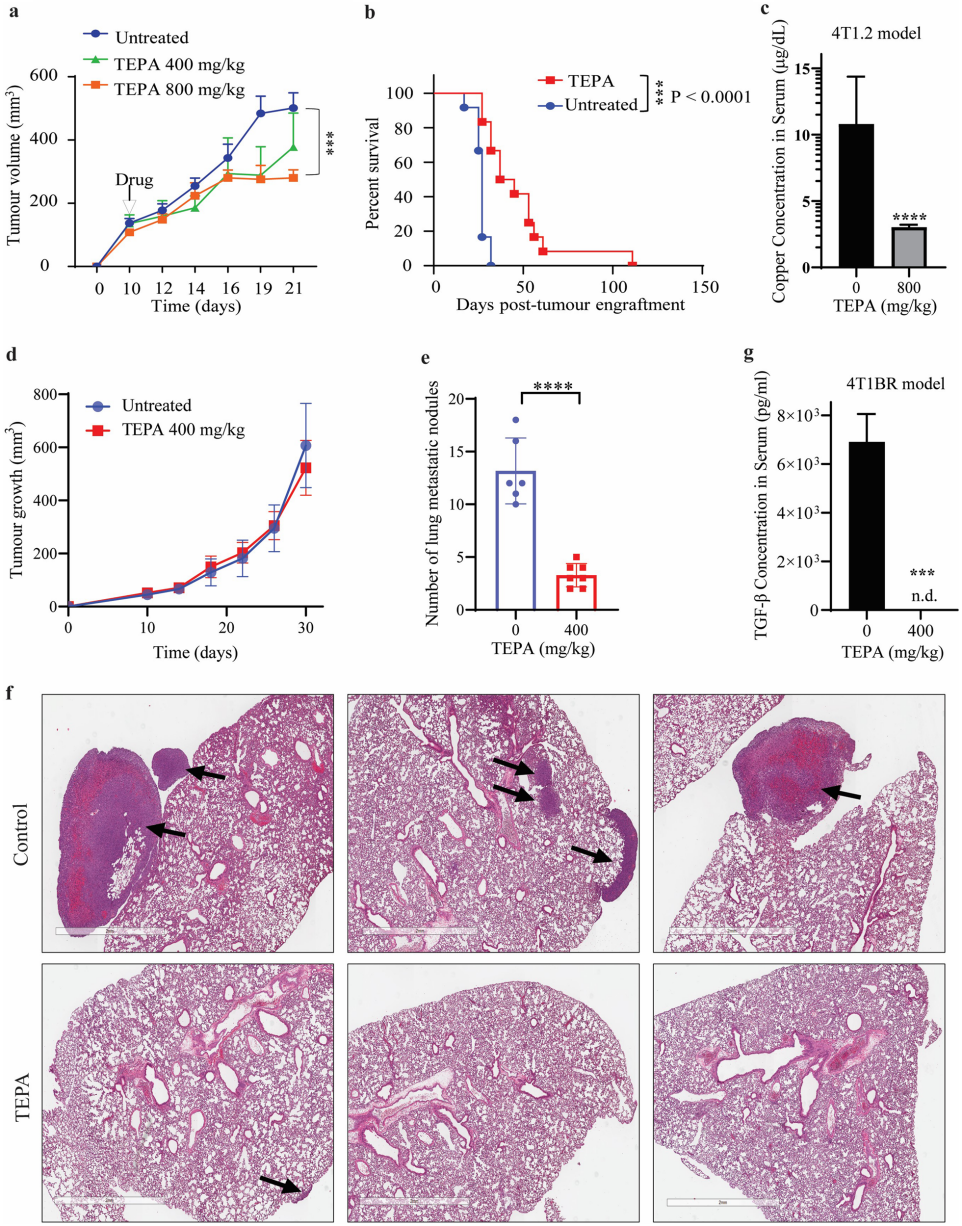
TEPA inhibits the in vivo tumor growth in a syngeneic model of triple-negative breast cancer. **a** Murine 4T1.2 syngeneic TNBC tumor growth following treatment with vehicle or TEPA (400 mg/kg and 800 mg/kg, daily, oral gavage) for three weeks is shown. Treatment was started once tumors became palpable (50–100 mm^3^). The mean tumor volume of each treatment group from each mouse is presented (n = 6 mice/group). Two-way ANOVA was followed by Sidak’s post-test for tumor growth analysis. **b** The survival analysis for murine 4T1.2 syngeneic TNBC tumors following treatment with vehicle or TEPA (800 mg/kg, daily, oral gavage) for three weeks is calculated by the Kaplan–Meier survival analysis. **c** Copper concentrations in 4T1.2 mice model sera treated with 800 mg/kg TEPA compared to the vehicle controls. Mice were treated with TEPA for three weeks, treatment was withdrawn, and mice were followed for survival until tumors reached 1000 mm^3^. Down, TEPA reduces lung metastases in 4T1BR metastatic TNBC model in vivo. Murine 4T1BR tumor-bearing mice were treated with vehicle or TEPA (400 mg/kg, daily, oral gavage) for three weeks. At the end of the treatment, lungs were isolated and stained with H&E to analyze tumor metastases. **d** Tumor growth analysis of murine 4T1BR tumor-bearing mice treated with vehicle or TEPA (400 mg/kg). Two-way ANOVA was followed by Sidak’s post-test for tumor growth analysis (n = 6). **e** Quantification of the number of metastatic lung nodules in the vehicle and TEPA-treated mice. Unpaired “t” test was performed (n = 6). **f** Representative images of lung metastasis in 4T1BR tumor model treated with the vehicle or TEPA. The metastatic nodules were stained with H&E. **g** The protein level of TGF-β in the sera of 4T1BR syngeneic mice treated with TEPA (400 mg/Kg, daily, oral gavage) for 30 days

We evaluated the anti-cancer activity of TEPA using a fully immunocompetent murine pre-clinical syngeneic model of TNBC. 4T1.2 is a highly aggressive murine TNBC that recapitulates human TNBC tumor phenotypes with high propensity for spontaneous metastasis. 4T1.2 cells were implanted into the 4th mammary fat pad of 6-weeks old female Balb/c mice, and once tumor size reached approximately 50 mm^3^, mice were treated with either vehicle (saline) or TEPA (400 mg/kg and 800 mg/kg) daily by oral gavage. We previously investigated the same concentrations of TEPA in our neuroblastoma mouse model [38]. We saw no major impact of TEPA 400 mg/kg dosage on primary tumor growth, whereas TEPA 800 mg/kg significantly inhibited the 4T1.2 tumor growth compared to vehicle-treated control mice (Fig. 1a). TEPA was well-tolerated in mice as evidenced by no significant change in body weight.

We next examined the effect of TEPA treatment on the overall survival of 4T1.2 tumor-bearing mice. 4T1.2 tumor-bearing mice were either treated with vehicle or 800 mg/kg TEPA. After 21 days, we withdrew the treatment and followed the mice for survival. We found that 800 mg/kg TEPA treatment significantly prolonged survival in the 4T1.2 tumor-bearing mice (Fig. 1b). The median survival time in the control mice was 27 days, where TEPA increased median survival to 41 days (p—0.0002). Moreover, immunohistochemistry of tumors from 4T1.2 mice revealed that E-cadherin, typical epithelial marker, was upregulated in the TEPA treated mice compared to the untreated controls (Additional file 1: Fig. S8). We also measured copper concentrations in 4T1.2 mice sera treated with 0 and 800 mg/kg TEPA. We found that 4T1.2 mice which had received TEPA had significantly lower copper levels in their sera compared to vehicle controls (Fig. 1c). Moreover, this experiment showed that TEPA at the 400 mg/kg dosage did not reduce the primary tumor, suggesting this could serve as an optimal dose to study the potential effect on EMT and metastasis in vivo.

### Copper depletion reduces lung metastases in 4T1BR metastatic TNBC model in vivo

Based on previous investigations which used copper chelator tetrathiomolybdate (TM) to reduce lung metastases in a TNBC model, we investigated the effect of TEPA on inhibiting metastases in vivo [7]. We investigated the effect of TEPA on lung metastasis using an aggressive syngeneic murine 4T1BR4 model for TNBC. The 4T1BR4 cells were derived from the parental 4T1 cells, however, they have a higher engraftment rate in the lungs [42]. We used TEPA at the reduced 400 mg/kg concentration, as this dose did not have a strong effect in reducing the tumor growth of the primary tumor (Fig. 1d). Our results showed a significant reduction of lung metastasis in lung nodules in mice treated with TEPA compared to their vehicle treated controls (P value > 0.001 Figs. 1e and 5f). This reduction in metastases was observed alongside monitoring of the primary tumor, which was not affected by the TEPA 400 mg/kg dose. Importantly, as confirmation of the ability of TEPA to target TGF-β signaling pathways, we observed a significant reduction of TGF-β levels in the serum of the mice treated with TEPA (Fig. 1g). We also confirmed this in the neuroblastoma TH-MYCN mouse model, where we observed decreased levels of TGF-β after treatment with the same concentration of TEPA (Additional file 1: Fig. S5). In vivo data confirmed the ability of TEPA to inhibit TGF-β signaling and reduce cancer metastasis.

### Copper chelation downregulates transcriptomic expression of EMT markers and transcription factors in cancer cell lines

**Fig. 2:**
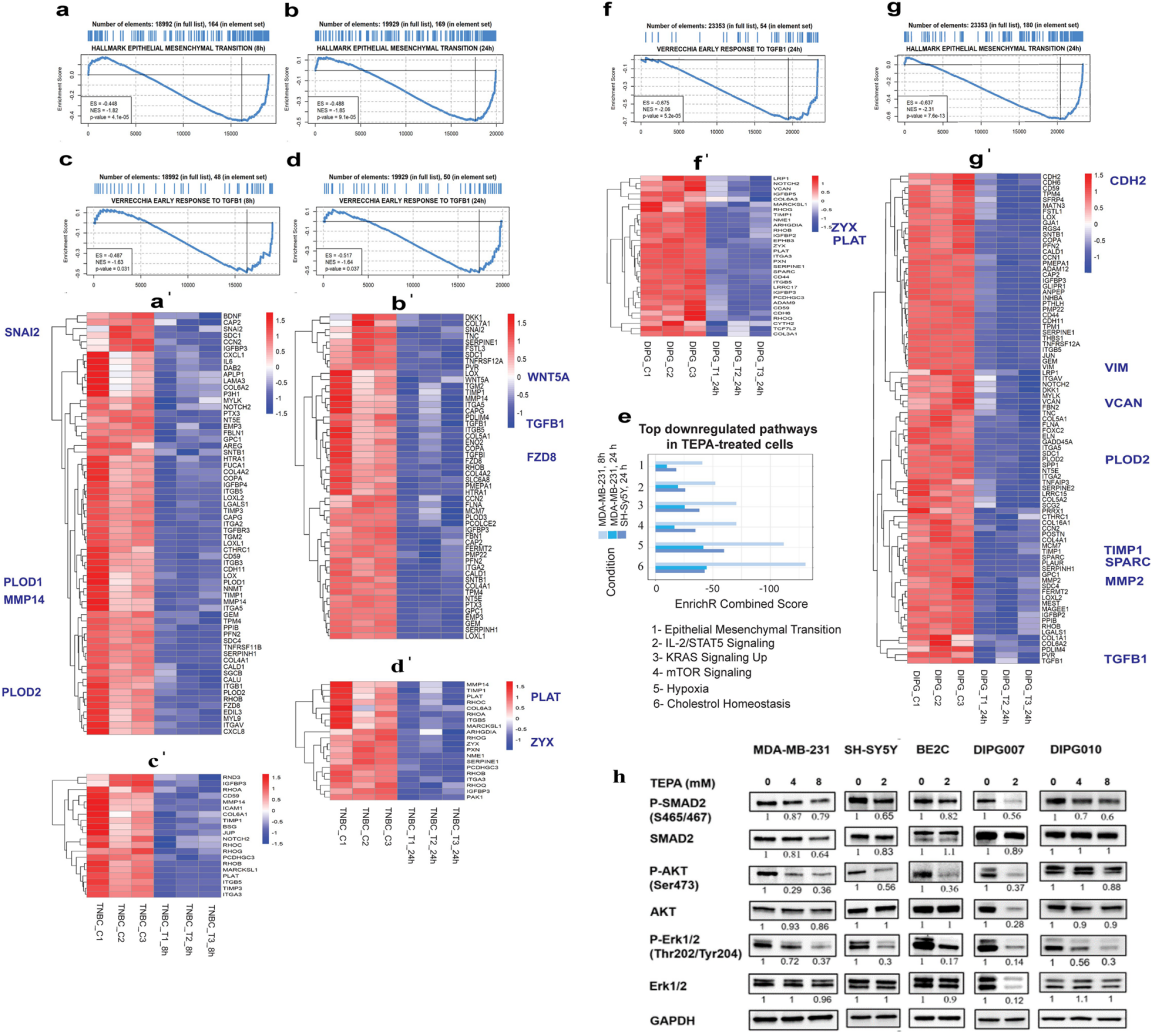
Enrichment analysis of TEPA-treated cells **a** and **b**, GSEA enrichment plot of EMT-related genes identified by RNA-Seq analysis in MDA-MB-231 cells treated with TEPA for 8 (left panel) and 24 (right panel) hours. Downregulation of the MSigDB Hallmark EMT gene set is consistently observed at both time-points. **a’** and **b’**, Heatmap showing EMT gene set leading-edge genes at MDA-MB-231 cells treated with TEPA for 8 (**a’**) and 24 (**b’**) hours. Heatmap color intensity is proportional to row-scaled VST-normalized expression.**c**, **d**, GSEA enrichment plot showing downregulation of TGFB1 early responsive genes identified by RNA-Seq analysis in MDA-MB-231 cells treated with TEPA for 8 (left panel) and 24 (right panel) hours. **c’**, **d’**, Heatmap showing MSigDB’s VERRECCHIA EARLY RESPONSE TO TGFB1 gene set leading-edge genes at MDA-MB-231 cells treated with TEPA for 8 (**c’**) and 24 (**d’**) hours. The heatmap color intensity is proportional to row-scaled VST-normalized expression. **e** Bar-plot showing common negatively enriched gene terms in MDA-MB-231 cells treated with TEPA for 8 and 24 h and SH-SY5Y treated with TEPA for 24 h. Enrichment analysis was performed with the enrich R package. A consistent downregulation of EMT and cholesterol metabolism can be observed in both breast cancer and neuroblastoma cells. Furthermore, downregulation of several signaling pathways (IL-2/STAT5, mTORC1 and KRAS) is observed in both cell models following TEPA treatment. **f** Up, GSEA enrichment plot of EMT-related genes identified by RNA-Seq analysis in DIPG010 cells treated with TEPA for 24 h. Downregulation of the MSigDB Hallmark EMT gene set is consistently observed at this time-point. **f’**, Heatmap showing EMT gene set leading-edge genes at DIPG010 cells treated with TEPA for 24 h. Heatmap color intensity is proportional to row-scaled VST-normalized expression. **g** Up, GSEA enrichment plot showing downregulation of early TGFB1 early responsive genes identified by RNA-Seq analysis in DIPG010 cells treated with TEPA for 24 h. **g’**, Heatmap showing MSigDB’s VERRECCHIA EARLY RESPONSE TO TGFB1 gene set leading-edge genes at DIPG010 cells treated with TEPA. The heatmap color intensity is proportional to row-scaled VST-normalized expression. **h** Analysis of TGF-β/SMAD2/3 and TGF-β/AKT/mTOR signaling pathways at protein levels. Western blot analysis of SMAD2, phospho-SMAD2, AKT, phospho-AKT, ERK1/2, and phosphor-ERK1/2 following treatment of MDA-MB-231, SH-SY5Y, BE2C, DIPG007, and DIPG010 cells with TEPA (0, 4 and 8 mM for MDA-MB-231 and DIPG010, and 0 and 2 mM for SH-SY5Y and DIPG007) for 24 h. GAPDH has been used as a positive and an endogenous control for normalization

To map the underlying molecular mechanisms by which the copper chelating agent (TEPA) inhibits EMT, we performed whole transcriptome analysis in TEPA treated TNBC (triple negative breast cancer), NB (neuroblastoma), and DIPG (diffuse intrinsic pontine glioma) cells (Fig. 2). Differential expression analysis comparing TEPA treated cells to untreated controls revealed that TEPA induced profound transcriptomic changes (Additional file 1: Fig. S2). A global Gene Set Enrichment Analysis (GSEA) revealed a consistent and significant downregulation of the Hallmark Epithelial to Mesenchymal Transition pathway after TEPA treatment. For example, in MDA-MB-231 cells, MMP-2, MMP-9, MMP-14, PLOD2, PLAT, MARCKSL1, CCR7, PAK1, and ZYX genes were downregulated and E-cadherin (CDH1), TIMP3 and TIMP4 were upregulated (Fig. 2a/a′ and b/b′, Additional file 1: Fig. S4a and Table S1). In the DIPG010 cell line, Vimentin (VIM), MMP-2, N-cadherin (CDH2), VCAN, PLOD2, ZYX and PLAT were downregulated (Fig. 2g/g′ & Table 1). Several crucial components of TGF-β signaling were also dysregulated by TEPA in these cell lines. In the MDA-MB-231 cell line, downregulation of lysyl oxidases, metallopeptidases MMP-11 and MMP-15, and TGFBR2, SNAI2, SPARC, RAC2, and BCL2, and upregulation of CDKN1A (Additional file 1: Table S1) was observed. In the DIPG010 cell line downregulation of SPARC, VIM, and TGF-β1 was observed. Interestingly, a time-dependent downregulation trend was detected for SNAI2 (also known as SLUG) in MDA-MB-231 TNBC cells. This is a well-studied transcriptional regulator of EMT, and its inhibition has been previously shown to decrease TNBC dissemination in animal models [43, 44]. Importantly, downregulation of the SNAI2 genes network was confirmed by Master Regulator Analysis (Additional file 1: Fig. S3). We also detected a significant downregulation of the VERRECHIA EARLY RESPONSE TO TGF-β1 pathway in TNBC (Fig. 2c/c′ and d/d′) and DIPG (Fig. 2f/f′) cell lines. Similarly, inhibition of TGF-β signaling and other EMT related pathways were observed upon TEPA treatment in NB cell lines (Fig. 2e).

**Table 1:** List of genes and proteins’ phosphorylation affected by TEPA in RNA-Seq, Real-Time PCR, and western blot analysis of MDA-MB-231, SH-SY5Y, DIPG007, and DIPG010 cell lines. These genes and proteins are important in the migration, and invasion of cancer cells via dysregulation of TGF-β and EMT pathways

Cell lines	Genes*I*protein symbol	
	Downregulated	Upregulated
MDA-MB-231	TGF-β1, TGFBR2, SPARC, SNAI2, SMAD2, p-SMAD2, MMP-2, MMP-9, MMP-14, MMP- 11, MMP-15, Vimentin, RAC2, BCL2, PLOD2, PLAT, MARCKSLl, CCR7, PAK l, ZYX, LAMA4, p-AKT, p-ERK l/2	CDKN1A, TIMP3, TIMP4, E-cadherin, (CDH l)
SH-SY5Y	TGF-β1, p-SMAD2, SMAD2, MMP-9, MMP-14, Vimentin, ZEBl, ZYX, RACl, PLAT, p-AKT, p-ERK l/2	TGIFl, CDKN1A
DIPG007	SMAD2, p-SMAD2, MMP-2, MMP-9, MMP-14, Vimentin, ZYX, VCAN, PXN, AKT, p-AKT, ERK l/2, p-ERK l/2	CDKN1C
DIPG010	TGF-β1, SPARC, N-cadherin (CDH2), Vimentin, MMP-2, ZYX, LAMA4, PLAT, VCAN	

In addition to EMT, we showed a significant negative enrichment of, hypoxia, cholesterol homeostasis, and IL-2/STAT5 pathways in the different tumor types (Fig. 2e).

### Copper homeostasis affects expression and cellular localization of EMT markers

**Fig. 3:**
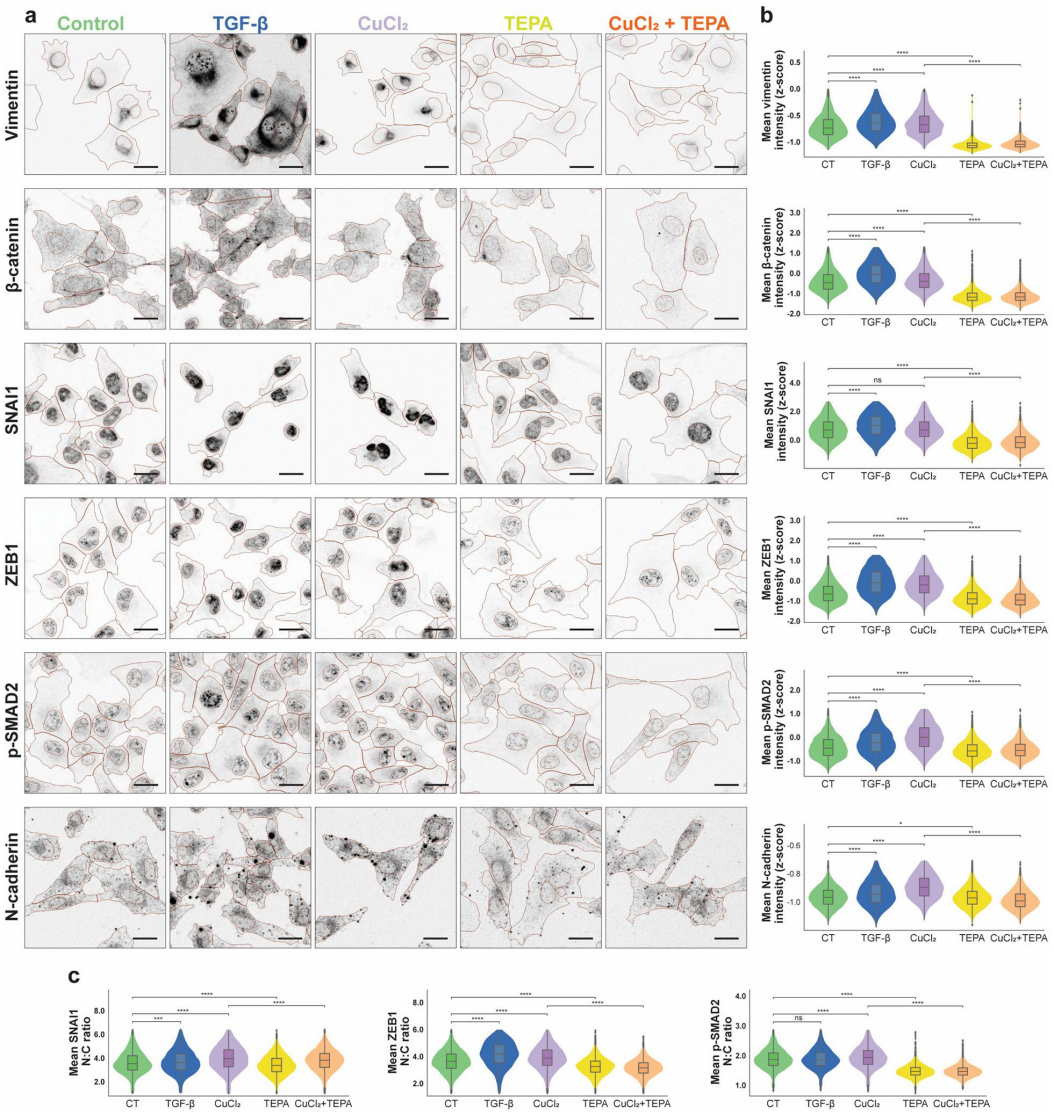
**a** Confocal images (inverted for signal visibility) showing epithelial and mesenchymal cell state markers in MDA-MB-231 TNBC cells across control or treated conditions (TGF- β, CuCl2, TEPA or CuCl2 + TEPA). Segmented cell nuclei and cytoplasm outlines shown (red). All scale bars 20 μm. **b** Violin plots showing quantification of single cell mean fluorescence intensities. **c** Violin plots showing quantification of single cell nuclear-to-cytoplasmic (N:C) mean intensity ratio. Total cells per treatment condition: control = 21848; TGF- β = 17743; CuCl2 = 19153; TEPA = 25200; and CuCl2 + TEPA = 18705. 14000–23000 cells per marker, 2000–6000 cells per marker per treatment group. Results of two-sided Mann–Whitney-Wilcoxon testing with Bonferroni correction: ns = (5.00e-02 < p <  = 1.00e + 00), *: (1.00e-02 < p <  = 5.00e-02), **: (1.00e-03 < p <  = 1.00e-02), ***: (1.00e-04 < p <  = 1.00e-03), ****: (p <  = 1.00e-04)

To investigate whether intracellular copper levels influence the expression of epithelial and mesenchymal (EM) state markers, we added copper to the media of MDA-MB-231 cells and used confocal microscopy to record single cells at a population scale (Fig. 3). Immunofluorescence labelling of mesenchymal protein markers VIM, β-Catenin, ZEB1, p-SMAD2 and N-cadherin were significantly increased by copper supplementation compared to untreated cells (Fig. 3a, b). This effect was completely abrogated by the addition of the copper chelator TEPA. Importantly, TEPA on its own caused a significant decrease in these mesenchymal markers compared to untreated control, suggesting endogenous intracellular copper could be further targeted to reduce the mesenchymal phenotype. Interestingly, unlike the other factors, SNAI1 protein did not increase significantly after copper supplementation, but was reduced by TEPA treatment. Perhaps this is because these cells already express a highly mesenchymal-like state, and SNAI1 protein endogenous levels are close to their maximum and cannot easily be increased. Importantly, for SNAI1, ZEB1 and p-SMAD2, confocal microscopy showed not only that the total level of these proteins decreased with TEPA treatment, but also that the nuclear: cytoplasmic localization ratio became more cytoplasmic after treatment (Fig. 3c), indicating suppression of their transcriptional role. As a positive control for mesenchymal modulation of the MDA-MB-231 cell type, we also treated cells with recombinant TGF-β. In accordance with TGF-β’s role in the promotion of EMT, all the mesenchymal markers analyzed displayed an increase in expression after TGF-β treatment, in almost all cases analogous to the effects of copper treatment. To further validate these results, we performed immunofluorescent staining for EMT markers in BE2C and SH-SY5Yneuroblastoma cell lines. Our data show that in BE2C cells, B-catenin, N-cadherin and Vimentin reduced 24 h after treatment with TEPA. Moreover, the nuclear levels of EMT transcription factors of pSMAD2 and SNAI1 reduced with TEPA. Furthermore, in SH-SY5Y, the expression of the mesenchymal markers Vimentin, N-cadherin and B-catenin were significantly reduced 24 h after adding TEPA (Additional file 1: Fig. S7).

**Fig. 4:**
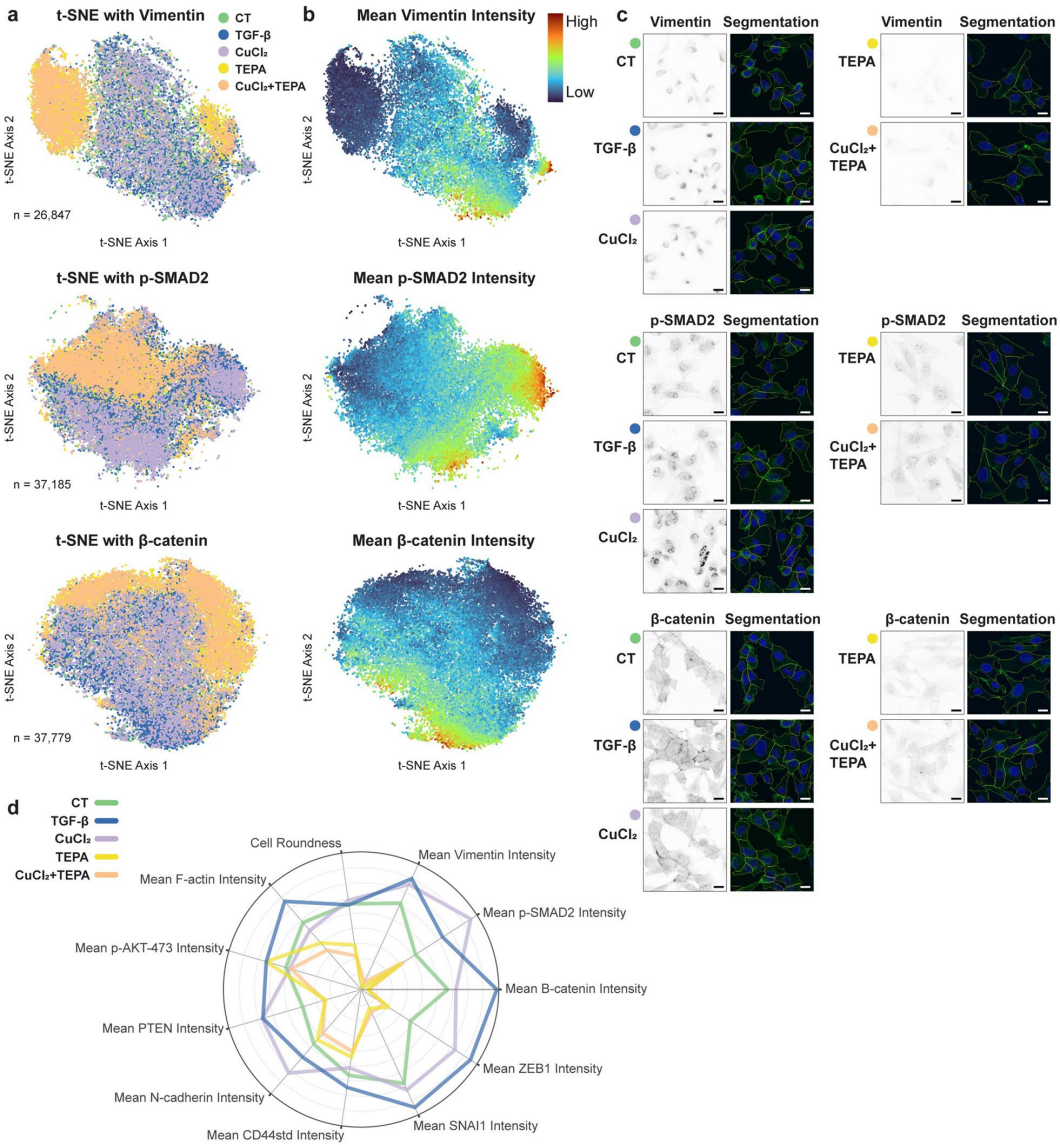
**a** Examples of multivariate single MDA-MB-231 TNBC cell phenotypic data depicted in t-distributed stochastic neighbor embedding (t-SNE) plots based on 728 quantitative features per cell capturing cell morphology, cell–cell contact levels, and per marker intensity features (levels, localization, textures) for DNA marker DAPI, F-actin marker phalloidin, and a third indicated epithelial or mesenchymal (EM) state marker (Vimentin or p-SMAD2 or b-catenin or E-cadherin). Color-coding reflects treatment condition (as indicated), with cell number (n) also shown. **b** Equivalent t-SNE plots color-coded by mean intensity per cell (expression level) of the indicated EM state marker. **c** Representative confocal image regions (inverted for signal visibility) of each EM state marker in each treatment condition, and matched confocal images of phalloidin (green) and DAPI (blue) labelling with cell body (yellow) and nuclear (magenta) segmentation boundaries shown. All scale bars 10 mm. **d** Radar plot contrasting average phenotypic signatures for all cells in each treatment condition (color coded as in **a**, **c**) across all [11] EM state marker intensities and [2] basic cell morphology features (Area, Roundness)

To explore in more detail the phenotypic effects induced by all of the above treatments, we analyzed the data extracted through confocal imaging and computational image analysis, now focused on a single cell level (Fig. 4). We assessed 728 quantitative image-derived features per cell to characterize cell phenotype based on cell morphology, cell–cell contact levels, nuclear marker (DAPI) and F-actin marker (phalloidin) in combinations with each of the EMT state markers detailed above. These features were statistically analyzed using t-distributed stochastic neighbor embedding (t-SNE) to visualize and compare phenotypes for at least 25,000 single cells per labeling condition. We observed strong segregation in phenotypic space between TEPA or copper + TEPA treated cells versus cells incubated with additional copper and TGF-β (Fig. 4a). This phenotypic segregation coincides with changes in per cell expression levels for each EM state marker as exemplified in Fig. 4b, c, but also incorporates changes in a huge range of additional cellular features. Combining data from all immunofluorescence labeling conditions (for all markers) enabled comparison of the average EM state ‘phenotypic signature’ associated with each of the five treatment conditions (Fig. 4c). The radar plot in Fig. 4 emphasizes the similarity in phenotypic signature between TEPA and copper + TEPA and between copper and TGF-β. More importantly there is a net strong distinction between cells supplemented with copper and TGF-β and the cells incubated with TEPA and TEPA + copper.

Taken together, this data demonstrates that both copper and TGF-β treatments induce highly analogous cell state changes consistent with a richly defined epithelial to mesenchymal transition. Copper chelation with TEPA suppressed the innate mesenchymal characteristics of MDA-MB-231 cells, and moreover, profoundly inhibited the potent EMT-inducing effects of TGF-β and copper supplementation.

### Copper depletion by TEPA inhibits EMT process by blocking TGF-β canonical and noncanonical signaling pathways

TGF-β is a well-known cytokine that acts on protein kinase receptors to induce a plethora of biological signals regulating EMT. To explore the potential effects of the copper chelator with TGF-β signaling, we interrogated our RNA-Seq data obtained from MDA-MB-231 (4 mM TEPA, for 8 and 24 h), SH-SY5Y (2 mM TEPA for 24 h) and DIPG010 (8 mM TEPA for 24 h) cancer cell lines. Our transcriptomics data showed that TGF-β canonical and non-canonical signaling pathways are dysregulated by TEPA in all three cancer types (Fig. 2 and Additional file 1: Fig. S6). Moreover, to better understand in detail which genes were early and late responders to TEPA treatment, we performed transcriptomics analysis in MDA-MB-231 after 8 h and 24 h of treatment. In Additional file 1: Table S1, we have summarized the signaling pathways and their specific genes, which are affected by TEPA treatment after 8- and 24- hours of incubation.

**Fig. 5:**
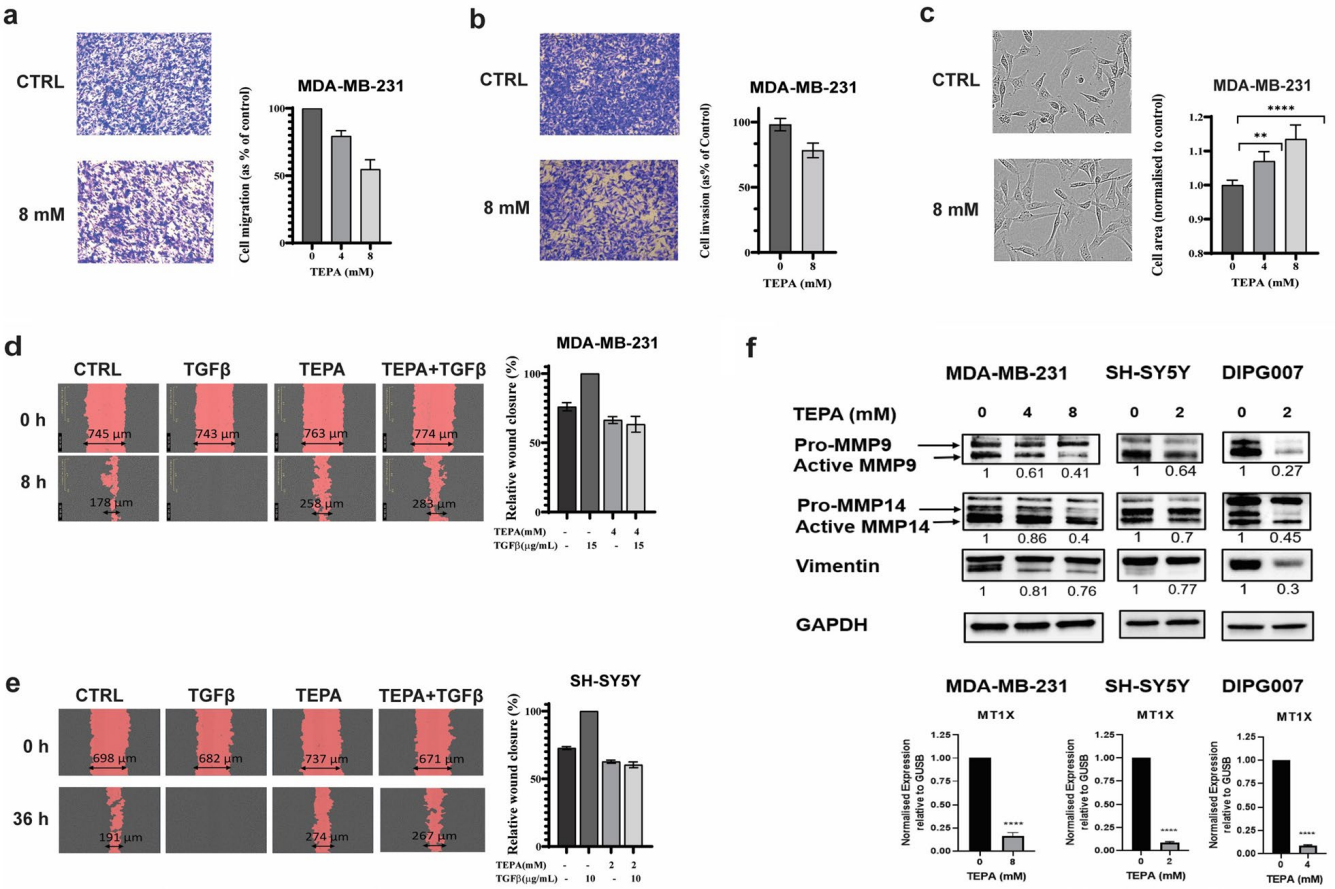
Inhibitory effect of TEPA on cell migration and morphology in TNBC (MDA-MB-231) and NB (SH-SY5Y) cells. **a** Representative image (n = 3) of transwell migration assays performed on MDA-MB-231 cells treated with 0, 4, and 8 mM of TEPA. **b** Transwell cell invasion assay for MDA-MB-231 cells treated with 0 and 8 mM of TEPA. **c** Representative morphology alteration of MDA-MB-231 cells treated with TEPA. Images show how TEPA treatment caused a transition from a mesenchymal to an epithelial-like phenotype. Images were taken at 100 × magnification using an ECLIPSE Ni-E light microscope. The graph shows the average cell size (µm2) relative to the control. Significance was determined by One-way ANOVA where ** and **** represent p < 0.001 and p < 0.0001, respectively. **d**, **e** Inhibitory effect of TEPA on cell migration induced by TGF-β in a scratch assay. After generating artificial gaps, TNBC and NB cells were pre-treated with TEPA (4 mM for TNBC and 2 mM for NB) for two hours and then with 15 ng/ml TGF-β for MDA-MB-231 and 10 ng/ml TGF-β for SH-SY5Y in complete media. Wound closure was monitored by IncuCyteS3 at 0 and 14 h for TNBC and 0 and 36 h for SH-SY5Y. Significance was determined by One-way ANOVA with p-value < 0.0001 for both MDA-MB-231 and SH-SY5Y cells. **f** Analysis of MMP-9, MMP-14, and vimentin at protein levels. Western blot analysis of MMP-9, MMP-14, and vimentin following treatment of MDA-MB-231, and SH-SY5Y, DIPG007, and DIPG010 cells with TEPA (0, 4 and 8 mM for MDA-MB-231 and DIPG010, and 0 & 2 mM for SH-SY5Y and DIPG007) for 24 h. GAPDH has been used as a positive and an endogenous control. Down, Expression analysis by quantitative PCR. The alteration of MT1X gene expression following treating MDA-MB-231, SH-SY5Y, and DIPG007 cells with TEPA for 24 h were analyzed by Real-Time PCR. MT1X is a surrogate marker which its expression is proportional to the intracellular copper concentration. GUSB was used as an internal control

To further validate our transcriptomics data, we performed western blot analyses in five cell lines from three different tumor types (Fig. 2h). In particular, we investigated the activity of TEPA on canonical TGF-β/SMAD2&3 and non-canonical TGF-β/PI3K/AKT, TGF-β/RAS/RAF/MEK/ERK1/2, and TGF-β/WNT/β-catenin signaling pathways. We found that TEPA downregulated the canonical TGF-β signaling pathway by reduction of both total SMAD2 expression and phosphorylation at its C-terminal domain (Fig. 2h). Next, we demonstrated the inhibitory effect of TEPA on the TGF-β non-canonical pathway by reducing AKT and ERK1/2 phosphorylation (Fig. 2h). Those results are consistent with our results obtained from the single-cell imaging experiments showing also that β-catenin levels and nuclear localization was downregulated by TEPA (Fig. 3). This inhibitory effect of TEPA on TGF-β is also evidenced by a significant decrease in VIM, MMP-9 and MMP-14, which play a key role in promoting tumor invasion (Fig. 5f). Moreover, western blot analysis demonstrated downregulation of MMP-2 in MDA-MB-231 and DIPG007 cells, and also reduced mTOR and phospho-mTOR proteins and an upregulation of E-cadherin in MDA-MB-231 cells after TEPA treatment (Additional file 1: Fig. S4a). Finally, as a demonstration of the ability of TEPA to reduce intracellular copper levels in cancer cells, we observed a robust reduction in MT1X expression, a metallothionine used as a surrogate measure of intracellular levels of copper, by real-time PCR (Fig. 5f). To confirm the results obtained by using the copper chelator TEPA, we treated BE2C cells with another clinically available copper chelator, (Tetrathiomolybdate). Our data confirmed a reduction of EMT markers and inhibition of both SMADs and non-SMADs signaling pathways occurs after copper chelation (Additional file 1: Fig S4b). Moreover, TGF-β cleavage and activation was also reduced after TM treatment (Additional file 1: Fig. S4c). Taken together, our data confirmed that copper chelation therapy significantly reduces EMT by downregulating TGF-β/pSMAD2, and TGF-β/PI3K/p-AKT, TGF-β/RAS/RAF/MEK/ERK, and TGF-β/WNT/β-catenin pathways in cancer cells.

### TEPA inhibits cancer cell migration and invasion

To investigate whether TEPA inhibits EMT-induced migration and invasion in cancer cells, we performed a migration assay on MDA-MB-231 cells by pre-treating them with TEPA. Cell migration was significantly inhibited (by 45%) by 8 mM TEPA compared to the non-treated control cells (Fig. 5a). This result was strengthened by additional data obtained from the transwell cell invasion assay showing the ability of TEPA to inhibit the invasion of cancer cells through the extracellular matrix, a critical process for metastasis initiation (Fig. 5b). Moreover, we found that chelating copper with TEPA alters the morphology and size of TNBC cells (Fig. 5c). Epithelial cells typically have a characteristic shape that is often described as polygonal or cuboidal. They are generally uniform in size and shape, with well-defined boundaries. Epithelial cells form strong intercellular connections through specialized junctions. In Fig. 4 we can see the presence of more of these junctions in the cells treated with TEPA, indicative of an epithelial phenotype. Moreover, in Fig. 5c, cells treated with TEPA are longer and they have the typical epithelial characteristics such as a moderate amount of cytoplasm. A scratch-wound healing assay in both MDA-MB-231 and SH-SY5Y cell lines were observed to have a reduction of cell migration when treated with copper chelating agent. Additional file 1: FigS.1a and 1b show that TEPA inhibited cell migration at non-toxic concentrations in both cell lines. Moreover, to understand if supplementation of TGF-β could accelerate cancer cell migration and whether TEPA would be able to counteract its effect, TGF-β was added to the cell culture media to accelerate the wound healing process in a scratch assay. It is important to highlight that TEPA maintained its inhibitory effect on cell migration in both TNBC and NB tumor cell lines, even after stimulation with TGF-β (Fig. 5d, e).

### Suggested mechanism of TEPA-mediated copper depletion in inhibiting EMT in cancer

**Fig. 6:**
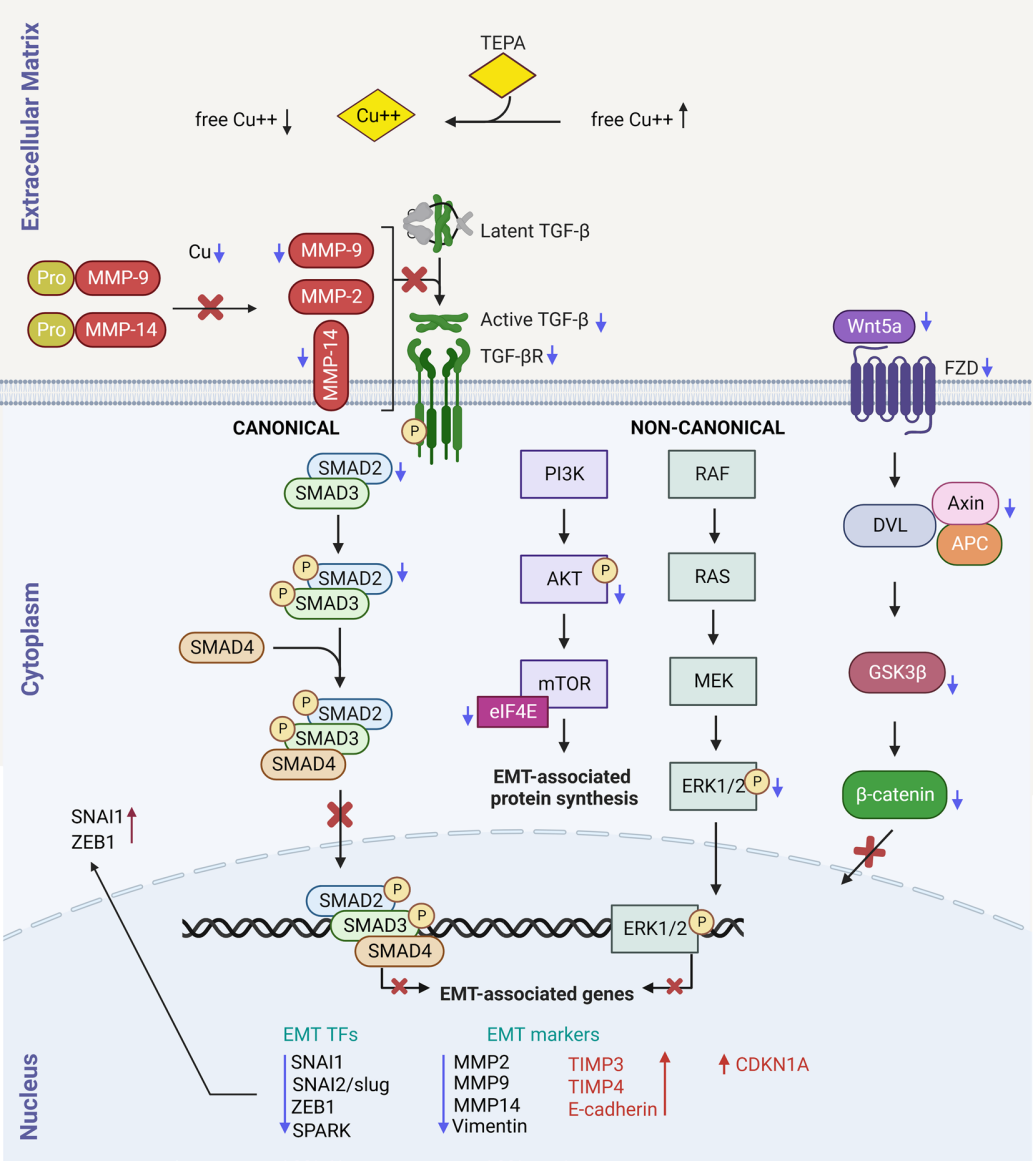
Molecular mechanism of tumor suppression by copper chelator therapy. TEPA has a regulatory effect on tumor invasion and metastasis by attenuating the canonical TGF-β/pSMAD2&3 and non-canonical TGF-β/AKT/mTOR, TGF-β/RAS/RAF/MEK/ERK, and TGF-β/WNT/β-catenin signaling pathways and downstream target genes related to EMT process in cancer initiation and progression. Blue and red arrows indicate downregulation and upregulation, respectively

TGF-β is a well-known cytokine that binds to protein kinase receptors to induce the EMT process. Our RNA-Seq data and experimental studies confirmed deregulation of TGF-β canonical (TGF-β/SMAD2&3) and non-canonical (PI3K/AKT/mTOR, RAS/RAF/ERK1&2, and WNT/β-catenin) signaling pathways with reduction of free copper levels (Fig. 6).

TEPA is a copper chelating agent that binds to Cu^2+^ at a 1:1 ratio and decreases extra- and intra-cellular levels of free copper in the cells. Here, we found that trapping copper not only downregulates MMP-2, MMP-9, and MMP-14 at the expression level but also decreases active forms of MMP-9 and MMP-14 proteins by inhibiting the cleavage of pro-MMP-9 and pro-MMP-14 metalloproteinases (Figs. 5f and 6). TEPA also increased the expression levels of MMP inhibitors such as TIMP3 and TIMP4 in TNBC (Additional file 1: Table S1) and TIMP1 in NB cells.

TGF-β is secreted into the extracellular matrix as an inactive dimeric form (latent TGF-β). Active MMP-9 and MMP-14 (cleaved forms) metalloproteinases cleave latent TGF-β and release the active form of TGF-β. TEPA reduces active TGF-β levels by downregulation of both MMP expression and cleavage (activation). Since active TGF-β levels were reduced by TEPA (Additional file 1: Fig. S5c and S5d), both canonical and non-canonical signaling pathways are affected (Fig. 6).

In the canonical TGF-β signaling pathway, expression of TGF-β (Additional file 1: Fig. S5b), TGFBR2 (Additional file 1: Table S1), and SMAD2 (Fig. 2h) are reduced after TEPA treatment. Moreover, in TNBC, TEPA blocks both phosphorylation and localization of p-SMAD2 into the nucleus and therefore affects the expression of crucial TGF-β and EMT-related markers such as MMP-2, MMP-9, MMP-14, vimentin, E-cadherin, TIMP3, TIMP4 and EMT-related transcription factors including SNAI1/snail, SNAI2/slug, SPARK, and ZEB1 (Figs. 2, 3, 5f and Additional file 1: Fig. S4 and S6). TEPA also affects SNAI1 and ZEB1 subcellular localizations by inducing their accumulation mostly in the cytoplasm, where they cannot function as transcription factors in the EMT process (Fig. 3).

In non-canonical TGF-β signaling, three molecular pathways including PI3K/AKT/mTOR, RAS/RAF/ERK1/2, and WNT/β-catenin pathways are inhibited by TEPA.

In PI3K/AKT/mTOR pathways, TEPA significantly decreased AKT phosphorylation in all studied cell lines (Fig. 2h), mTOR protein, phospho-mTOR (Additional file 1: Fig. S4) and eIF4E (Additional file 1: Fig. S6). eIF4E is one of the main mTOR targets and has crucial roles in the recruitment of ribosomes and translation initiation, both of which were downregulated in TNBC cells. Additionally, TEPA upregulated PTEN (a negative regulator of PI3K) 24 h after treatment in the TNBC cells (Additional file 1: Fig. S6).

TEPA downregulated the RAS/RAF/ERK1/2 signaling pathway by inhibiting ERK1/2 phosphorylation in TNBC (MDA-MB-231), NB (SH-SY5Y & BE2C), and DIPG cells (DIPG007 & DIPG010) (Fig. 2h). Moreover, TIAM1 and RASSF5 genes were downregulated in TNBC at RNA levels (Additional file 1: Fig. S6).

Finally, TEPA affected the WNT/β-catenin signaling pathway by downregulating β-catenin (Fig. 3) and WNT ligands (WNT5a, WNT7b, WNT9a, and WNT10b), FZD receptors (FZD1 & FZD8), and Axin2 (Additional file 1: Table S1) in TNBC cells. We also previously showed that GSK3β phosphorylation was downregulated by TEPA in NB cells [45]. Moreover, we found that the nucleus:cytoplasm ratio of β-catenin was reduced after treatment (Fig. 3).

Taken together, our results suggest that the potential mechanism of action of copper depletion by TEPA in inhibiting EMT process is caused by the downregulation of TGF-β/SMAD2&3, PI3K/AKT/mTOR, RAS/RAF/MEK/ERK1/2, and WNT/β-catenin signaling pathways at the genomic and proteomic levels.

## Discussion

Copper is a crucial metal which acts as a co-factor for many metalloenzymes, and proteins involved in pathways frequently dysregulated in cancer. As many of these pathways are dysregulated across multiple cancers, copper promotes tumor progression in a wide variety of tumors, including breast, lung, colon and melanoma cancers. Copper chelation has shown efficacy in several cancer types, and it was recently proposed as a potential adjuvant strategy to overcome BRAF-MEK1/2 inhibition gained resistance in melanoma and colon cancer [46, 47]. We previously reported that the copper chelator TEPA was able to reduce tumor growth and PD-L1 expression which increased immune cell infiltration into the tumor microenvironment in the TH-MYCN neuroblastoma mouse model [45]. In the current study, we found that treatment of the 4T1BR TNBC mouse model with a concentration of TEPA which did not have an effect on reducing the primary tumor size, was able to reduce lung metastasis.

A recent pre-clinical study has shown that copper chelation therapy is effective in preventing metastasis in mice with triple-negative breast cancer. Though the molecular underpinnings of that mechanism were largely unexplored, strong evidence indicating anti-angiogenic effects were highlighted. Our study provides further evidence of the anti-metastatic efficacy of copper chelators focusing on molecular mechanisms. Copper chelators are established to have multiple mechanisms of action, and we aimed to identify the metastatic mechanisms which may be targeted in multiple cancer types. Using transcriptomic data from three different tumor types and additional supporting studies, we determined copper chelation inhibits the EMT process via downregulation of TGF-β signaling pathways in cancer.

EMT is a reversible process in which epithelial cells lose their cellular properties towards a more mesenchymal phenotype, allowing invasion, cell migration, tumor development, and metastasis [48, 49]. TGF-β exerts its effect on promoting EMT via canonical (SMADs) and non-canonical (PI3K/AKT/mTOR, RAF/RAS/MEK/ERK & WNT/β-catenin) TGF-β signaling pathways. In this study, we have demonstrated that copper chelation can inhibit epithelial-mesenchymal transition and shift cell properties from a mesenchymal state to a more epithelial-like phenotype in TNBC, NB and DIPG cancer cells via attenuating TGF-β signaling pathway activation and impair tumor invasiveness. Diminished intracellular copper availability influenced both canonical and non-canonical TGF-β signaling pathways, perturbing the expression of several key genes involved in EMT such as TGF-β1, MMP-2, MMP-9, MMP-14, E-cadherin, VIM, ZYX, VCAN, ITGAs, as well as crucial transcription factors responsible for the mesenchymal fate (SNAI2/SLUG, SNAI1/snail, ZEB1, and SPARC). Interestingly copper depletion changed the location of EMT markers SNAI1 and ZEB1, which are important transcription factors in the EMT process. Their shifted localization to the cytoplasm would suggest their transactivation activity on DNA is reduced. These changes to EMT markers were functionally validated by our wound healing and migration assay results, with both TNBC and NB cell lines observed to have decreased migration with TEPA treatment.

TGF-β is usually secreted into the extracellular matrix in an inactive form (latent TGF-β), where active MMP-9 and MMP-14 cleave latent TGF-β and release the active form of TGF-β in the extracellular matrix, triggering EMT processes [50–55]. Copper chelation with TEPA decreased active forms of MMP-9 and MMP-14 in MDA-MB-231, SH-SY5Y, and DIPG007 and MMP-2 in MDA-MB-231 and DIPG007 cells, exerting an alternative level of control on TGF-β function in addition to direct attenuation of TGF-β transcription. Additionally, TEPA significantly increased the expression of TIMP3 and TIMP4 in TNBC, and TIMP1 in NB cells 24 h after treatment. TIMP1, TIMP3, and TIMP4 are important inhibitors of MMP-9 protein [56, 57]. TIMP proteins bind to the active site of MMPs and inhibit their activity by blocking binding of MMPs to ECM substrates [58].

As copper is an essential element for cancer cells, lowering copper levels also induced downregulation of a broad panel of genes whose products impact many critical cancer-related signaling pathways, as depicted in Additional file 1: Table S1, including apoptosis, angiogenesis, and inflammation by reducing BCL2 and VEGF protein expression, respectively, and interestingly collagen production. Investigating each of these pathways in future provides further mechanisms and justification for the clinical use of these agents.

When we analyzed the cytokine profile and RNA-Seq data from NB, TNBC and DIPG cell lines, TGF-β was significantly reduced in all cancer types as were the EMT-related pathways. It is interesting that TGF-β is reported to play an important role in cancer immune evasion [59]. TGF-β suppresses the immune system by increasing PDL1 expression, which is dependent mainly on the activation of PI3K/AKT pathway [60]. This action is consistent with our previous finding in neuroblastoma that copper chelation altered tumor immune evasion and reduced PDL1 expression [45]. Transcriptomic data from TEPA treated breast cancer cells also displays changes in the immune-related genes (including IL6, IL6R, IL15, CXCL1, CXCL8, and PTX3) in breast cancer. Future studies are needed to ascertain if copper chelation inhibition of TGF-β signaling is required to alter the immune evasion previously reported in the neuroblastoma context or even as a general anti-cancer mechanism. This aspect may have led to improved immune surveillance and subsequently contributed to inhibition of metastatic spread in our in vivo model and may indicate that copper chelation could be used in place of, or in combination with immunomodulation cancer therapy regimes.

Many drugs targeting TGF-β have failed in clinical trials, partly because of severe side effects arising in patients [13–17]. This may raise concerns over the translational feasibility of the therapies directly targeting TGF-β. This is not the case of copper chelation therapy as it has very low toxicity. In fact, the clinical approved analogue of TEPA, called Trientine, has been safely used in both pediatric and adult patients with Wilson’s disease for decades.

## Conclusion

In conclusion, we report for the first time that reducing copper levels in cancer cells causes depletion of TGF-β, which results in the inhibition of EMT and metastasis. In consideration of the fact that copper chelating agents are already used for Wilson’s disease, are non-toxic and can deplete copper from all tissues of the body, this study provides an attractive framework for a new therapeutic strategy to target TGF-β and subsequently reducing both tumor growth and metastatic potential.

## Methods

### In vivo xenografts and tumor growth analysis

All experiments were approved by the QIMR Berghofer Medical Research Institute Animal Ethics Committee and performed as per the committee’s guidelines [61]. For murine 4T1.2 and 4T1BR4 TNBC syngeneic models, 1 × 10^5^ cells were prepared in PBS and injected into the right 4^th^ inguinal mammary fat pad of 6-weeks old female immunocompetent Balb/c mice. Once tumor size reached 50 mm^3^, mice were randomized blindly into different treatment groups and were then treated with the vehicle (saline) or TEPA. Two treatment groups were created, one was treated with 400 mg/kg of TEPA and the second with 800 mg/kg of TEPA as daily, oral gavage which was continued for three weeks. Tumor growth was measured thrice weekly using a digital caliper. Tumor volume was measured using the following formula: tumor volume = [L × W^2^]/2, where W = width of the tumor and L = length of the tumor.

### In vivo lung metastases analysis

4T1BR cells were injected into 6-weeks old female Balb/c mice as described above. Once tumor size reached ~ 30–50 mm^3^, mice were randomized blindly into different treatment groups and were then treated with the vehicle (saline) or TEPA (400 mg/kg, oral gavage, daily) for three weeks. This concentration was used as it did not impact the primary tumor growth in this model. At the end of the treatment, all mice were euthanized, and their lungs were harvested. For lung metastasis, lungs were perfused with 1X PBS to remove residual blood; then they were fixed with 10% formalin and stained with hematoxylin and eosin (H&E) to facilitate the counting of micro- and macro-metastatic nodules.

### Measurements of TGF-β in mice sera.

The detection of TGF-β in the cell culture media from SH-SY5Y and MDA-MB-231 cells treated with TEPA for 24 h was performed according to the protocol suggested by Thermo Fisher (TGF-beta-1-Human-ELISA-Kit/BMS249-4).

For the in vivo experiments in the 4T1.2 syngeneic model, whole blood was obtained from the tail vein of mice 30 days after commencing treatment with TEPA 800 mg/kg. Serum was obtained through centrifugation (15 min at 2500 RPM) of clotted blood and stored at − 70 °C until use. TGF-β levels (pg/mL) were determined using a commercial murine ELISA kit (#BMA608-4; Invitrogen, Massachusetts, USA) according to the manufacturer’s instructions. Samples were assayed in duplicate and expressed as the average reading using a Benchmark Plus microplate spectrophotometer reader (Bio-Rad, California, USA) at 450 nm (620 nm reference wavelength). To measure TGF-β in Th-MYCN neuroblastoma model, we used sera collected from mice treated with TEPA (400 mg/kg) obtained from our previous study [45].

### Cell lines and treatments

The human triple-negative breast cancer cell line, MDA-MB-231, was kindly provided by Associate Professor Caroline Ford from UNSW (Sydney, Australia). Neuroblastoma cell line, SH-SY5Y (SY5Y) was sourced from ATCC with Loretta Lau. SH- SY5Y and MDA-MB-231 cells were cultured in DMEM, containing 2 mM L-glutamine (Gibco, Massachusetts, USA) and 10% fetal bovine serum (FBS) (Sigma, Massachusetts, USA) and incubated in a humidified incubator with 5% CO_2_ at 37 °C. HSJD-DIPG007 (DIPG007) cells were kindly provided by Dr. Angel Montero from the Hospital Sant Joan de Die in Spain. VUMC-DIPG010 (DIPG010) cells were kindly provided by Dr Esther Hulleman from the VU University Medical Centre in the Netherlands. Both DIPG lines were grown in a cancer stem cell media and growth factors [62], specifically a 1:1 mix of D-MEM/F-12 with Neurobasal-A Medium (Gibco, Massachusetts, USA) supplemented with HEPES Buffer Solution (10 mM) (Gibco, Massachusetts, USA), Sodium Pyruvate (1 mM) (Gibco, Massachusetts, USA), 1% MEM non-essential amino acids solution (Gibco, Massachusetts, USA), 1% GlutaMAX-I (Gibco, Massachusetts, USA), 1% antibiotic–antimycotic (Gibco, Massachusetts, USA), B27 supplement minus vitamin A (Gibco, Massachusetts, USA), human FGF (20 ng/mL), human EGF (20 ng/mL), human PDGF-AA (10 ng/mL), human PDGF-BB (10 ng/mL) and heparin solution (2 µg/mL) and 10% FCS to promote adherent cell growth. All the treatments for RNA-seq, Real-Time PCR and western blots have done with 0, 4 & 8 mM TEPA for MDA-MB-231 and DIPG010, and 0 & 2 mM TEPA for BE2C, SH-SY5Y, and DIPG007 for 24 h.

### Reagents

Triethylenetetramine dihydrochloride (TEPA) was purchased from Sigma-Aldrich (Cat. No. T5033-25G). Stock solution for in vitro work was prepared by dissolving the powder in complete cell culture media. For in vivo studies, TEPA was prepared in saline and administered by oral gavage. Ammonium tetrathiomolybdate (TM) was purchased from Merck (Product No. 323446).

### Immunofluorescence staining

MDA-MB-231 cells were seeded as 4000 cells/well in 96-well plate for an overnight and treated with 20 µM CuCL2, 15 ng/mL TGF-β1, and 4 mM TEPA in different conditions for 24 h. Then, cells were fixed with 4% paraformaldehyde (Emgrid Australia, Cat# 15710) for 15 min and permeabilized with 0.1% Triton X-100 for 15 min at room temperature (RT). Following permeabilization, cells were blocked with Intercept (TBS) Blocking Buffer (LI-COR, Cat# LCR-927-60001) and incubated for 30 min at RT with the following primary antibodies: N-cadherin, vimentin, SNAI1phospho-AKT (Ser473), β-catenin, ZEB1, phospho-SMAD2. Cells were then washed four times with Intercept Blocking Buffer, followed by a 15 min incubation with the secondary antibodies: anti-mouse AlexaFluor 488 and anti-rabbit AlexaFluor 555. Cell nuclei and actin filaments were stained simultaneously with 4′,6-Diamidino-2-phenylindole dihydrochloride (DAPI) and Phalloidin-Atto 647N respectively. Before imaging, cells were washed four times with 1 × TRIS buffered saline. The information and dilution of used antibodies has been mentioned in Additional file 1: Table S2.

### Microscopy

Images were acquired using a Nikon AX R confocal microscope (Nikon, Tokyo, Japan) equipped with a 20 × Plan Apochromat air objective (NA 0.75) at 2 × zoom and resonant scanning at 2048 × 2048 resolution. 5 z-planes with 600 nm z-spacing were acquired per field and a maximum intensity projection was computed and used for subsequent image analysis steps. Channels were acquired in series with three passes: 405 nm and 647 nm in pass 1, 488 nm in pass 2 and 555 nm in pass 3.

### Image and data analysis

CellProfiler1 (Broad Institute, MIT, Massachusetts, USA) with Cellpose2 module integration was used for image analysis and quantitative feature extraction. Cell nuclei were detected using a custom Cellpose model3 trained on DAPI images. DAPI and Phalloidin images were combined and used to train a second custom Cellpose model for the detection of cell bodies. Generated nuclei masks were subtracted from the cell body masks to produce cytoplasm masks. Mean total, nuclear, and cytoplasmic marker intensities were extracted for each cell. In addition, a total of 728 quantitative features were extracted per cell defining cell morphology (size and shape features), cell–cell contact features, and intensity features characterizing levels, localization and texture of each marker present, per cell.

Single cell data was parsed and analyzed using a custom-developed Knime4 workflow (KNIME AG, Zurich). Per-cell nuclear-to-cytoplasmic (N:C) ratios were calculated by dividing the mean nuclear intensity by the mean cytoplasmic intensity for each marker. Outlier cells were detected and removed by means of interquartile range, R = [Q1–k(IQR), Q3 + k(IQR)] with IQR = Q3–Q1 and k = 1.5, using mean cell intensity and N:C ratio of each marker. Mean cell intensities were normalized via z-score. Pairwise Wilcoxon Rank Sum Tests were performed to compare fluorescence intensities between treatment groups.

For t-distributed stochastic neighbor (t-SNE) embedding [63], all (728) per cell features were normalized. Dimension reduction via principal component analysis (PCA) preceded t-SNE calculation. t-SNE scatter plots were generated using the ggplot2 R package [64]. Radar plots were generated using the Plotly.js function in Knime.

### Cell migration assay

Cell migration was assessed using the QCM Chemotaxis Cell Migration Colorimetric Assay kit (ECM506, Merck, USA) as the manufacturer instructed. Briefly, MDA-MB-231 cells (1.5 × 10^5^ cells/well) were seeded in 6-well plate and incubated at 37 ˚C for 4 h. Then, MDA-MB-231 cells were treated with 0, 4, and 8 mM TEPA for 24 h. Then, the cells were harvested and seeded into the top chamber of the inserts containing low serum Media (0.5% FBS). The bottom chamber included media with chemoattractant (20% FBS) and corresponding TEPA concentrations that were consistent with the upper chambers. The migration plates were incubated in a humidified incubator with 5% CO_2_ at 37 °C for 16 h. At the endpoint, transwell inserts were stained with crystal violet and imaged at 100 × magnification using Nikon Eclipse microscopy (Molecular Devices, California, USA).

### Real-time monitoring of wound healing cell migration using IncuCyte imaging

MDA-MB-231 (2 × 10^4^ cells/well) and SH-SY5Y cells (4 × 10^4^ cells/well) were seeded in 96-well plates and cultured overnight to allow the cells reach confluency. The next day, an artificial gap was generated on a confluent monolayer of cells using an Incucyte® 96-well Woundmaker Tool (Satorious, Germany) and cells were washed twice with PBS. Cells were then treated with specific amounts of TEPA (0, 4, and 8 mM for MDA-MB-231, and 0 and 2 mM for SH-SY5Y). For the co-treatment experiment, TGF-β was used at 15 ng/mL for MDA-MB-231, and at 10 ng/mL for SH-SY5Y cells. After treatment plates were immediately incubated at 37˚C, 5% CO_2_. Cells were imaged every 2–4 h. Images and data were obtained using IncuCyteS3 (Essen Bioscience) with a 10 × object. Incucyte® Scratch Wound Analysis Software Module (Cat. No. 9600–0012) was used to automatically measure the scratch length.

### RNA sequencing

MDA-MB-231 cells were plated at 5 × 10^6^ cells/T75 flask in DMEM media supplemented with 10% FBS and treated with 4 mM TEPA for 8 and 24 h. DIPG010 cells were seeded in 6 well plates at 5 × 10^5^ cells/well and treated with 4 mM TEPA for 24 h. Cells were then harvested, washed with PBS, and lysed using the Qiagen RNA Extraction Kit (RNEasy kit, catalog no. 74104, Qiagen, Hilden, Germany) following the recommended protocol. RNA purity and concentration were assessed using the Nanodrop 2000 (Thermo Fisher Scientific, USA). Quality control and library preparation were carried out by Ramaciotti Centre for Genomics (UNSW, Australia). Paired-end 100-bp sequencing was performed on the Illumina NovaSeq 6000 using TruSeq stranded mRNA. The data have been deposited under Gene Expression Omnibus (GEO) accession number GSE185760. We also analyzed our RNA-Seq data obtained from SH-SY5Y treated with TEPA (accession number: GSE155031).

### Bioinformatics analysis

After a quality check performed with FastQC [65], paired-end RNA-Seq data were aligned to the human genome assembly (build hg38) with the HISAT2 alignment program [66]. Raw gene counts were obtained by running featureCounts [67] with the following settings: -p -s 2 -t exon -g gene_name. Reads were mapped to genomic features using Homo_sapiens.GRCh38.103.gtf [68]. Differential expression analysis was performed using DESeq2 (v1.32.0) [69] in R [70]. Enrichment analysis was performed using the enrichR package (v3.0) [71], while Gene Set Enrichment Analysis (GSEA) was performed using fgsea (v1.18.10) R package and the human gene sets from the Broad Instituteˈs MSigDB, accessed through the msigdbr package (v7.4.1) [72–74]. Enrichment plots were generated using plotting functions from the corto package (1.1.8) [75]. The Gene expression matrix was obtained by applying Variance Stabilizing Transformation (VST) to raw counts data before plotting [76]. SH-SY5Y expression matrix was obtained from GEO (accession number: GSE155031) [40].

### Western blot analysis

TNBC and NB cell lines (2 × 10^5^ cells/well) and DIPG cells (5 × 10^5^ cells/well) were seeded in 6-well plates overnight and then were treated with specific amounts of TEPA (0, 4, and 8 mM for MDA-MB-231, and 0 and 2 mM for SH-SY5Y and DIPG007), and 0, 4 and 8 mM for DIPG010. After 24 h, cells were harvested and lysed in cold RIPA lysis buffer supplemented with 1X cOmplete Mini, EDTA-free Protease Inhibitor Cocktail, and PhosSTOP™ phosphatase inhibitor (both Sigma-Aldrich, Schnelldorf, Germany). Protein content was measured using BCA Protein Assay Kit (Thermo Fisher Scientific, USA). An equal amount of protein from control and each experiment was loaded onto 4–20% SDS-PAGE gels (Mini-PROTEAN TGX Gels, Bio-Rad, USA) and transferred to the nitrocellulose membranes (Amersham™ Protran^®^ 0.45 µM, GE Healthcare, Germany). Nitrocellulose membranes were blocked in 5% BSA/TBS-T (1X TBS + 0.1% Tween-20) for 1 h and probed with antibodies against studied proteins for an overnight at 4 ˚C. After washing in TBS-T, membranes were incubated for 1 h at room temperature with anti-rabbit (1:5000) or anti-mouse (1:5000) IgG secondary antibodies. The resultant immunoreactive band signal was visualized using Clarity ™ Western ECL Substrate (Bio-Rad, USA). The accurate quantification of the relative density between samples was analyzed by Image Lab™ software v6.0 (Bio-Rad, California, USA) and normalized with GAPDH, as a housekeeping protein. A list of antibodies used for mentioned proteins and their information is mentioned in Additional file 1: Table S3.

### RNA extraction and quantitative PCR (qPCR)

TNBC, DIPG and NB cell lines were seeded and treated as previously described in the western blot section. Total RNA was extracted using the RNeasy Mini Kit (#74106; QIAGEN, California, USA) according to the manufacturer’s instructions and its quality and concentration was assessed using the Nanodrop 2000 (Thermo Fisher Scientific, USA). First strand cDNA was synthesized with 1 µg of total RNA using SuperScript II Reverse Transcriptase (Invitrogen, Massachusetts, USA) according to the manufacturer's instructions. 40 ng of the resulting cDNA was used in the subsequent qPCR analysis using SsoAdvanced Universal SYBR® Green Supermix (#1725270) and CFX96 Real-Time PCR Detection System (both Bio-Rad, USA) with the following PCR program: 95 °C for 3 min, followed by 40 cycles of 95 °C for 10 s, 60 °C for 30 s and 72 °C for 30 s). Primer sequences were as follows: *MT1X* (F: GCTTCTCCTTGCCTCGAAAT and R: GCAGCAGCTCTTCTTGCAG), *TGF-β* (F: AAGTGGACATCAACGGGTTC and R: GTCCTTGCGGAAGTCAATGT) and *GUSB* (F: TGGTGCGTAGGGACAAGAAC and R: CCAAGGATTTGGTGTGAGCG). Quantifications were normalized using *GUSB* as the endogenous control.

### Statistical analysis

Statistically significant differences were determined if the p-value by an ordinary one-way ANOVA and paired t-test was less than or equal to 0.05 (Prism version 8.3.1).

## Supplementary Information

**Additional file1: Figure S1.** Analysis of cell migration following treating cells with TEPA. **a & b** Cell migration analysis by scratch wound assay for MDA-MB-231 and SH-SY5Y cells, respectively. Pink areas indicate the scratch-wound area. This experiment have done as triplicate and significance was confirmed by p-value <0.001. **Figure S2.** Gene expression changes in TEPA-treated MDA-MB-231 cells. Volcano plots showing gene expression changes after 8 (**a**) and 24 (**b**) hours of TEPA treatment. The top significant 25 up and top 25 down-regulated genes are labeled. **c** K-means clustering classifies gene expression changes into 4 clusters. Genes included in cluster 4 show a time-dependent downregulation. **d** Top 10 MSigDB Hallmark enriched gene sets. Significant enrichments of MSigDB gene sets were evaluated through enrichment analysis performed with EnrichR. Color intensity is referred to the enrichment score computed by EnrichR and calculated as follows: combined score = log(p) * z, where p is the Fisher exact test p-value, and z is the z-score for deviation from expected rank. Asterisks mean a corrected p-value for multiple testing < 0.05. **Figure S3. Master Regulator Analysis. **SNAI2 sub-network downregulation in TEPA-treated cells at 8 (up) and 24 hours (down). The top 12 highest-likelihood targets are shown on the right side. The genes in each network are shown in a barcode-like diagram showing all transcriptome genes by means of their differential expression upon TEPA treatment, from the most downregulated (left) to the most upregulated(right). A blue background on the NES box is used to indicate a negative enrichment (or repression of the corresponding co-expression network). **Figure S4. a** Western blot analysis of MMP-2 in cell supernatant, and mTOR, phospho-mTOR (Ser2448), and E-cadherin (CDH1) in cell lysate. For MMP-2, cells were treated with specific amount of TEPA in serum-free media for 24 hours. Then cell supernatants were collected, and soluble proteins were concentrated using Ultracel-10 regenerated cellulose membrane (Amicon® Ultra-15 Centrifugal Filter Unit, Merck). 20 µg of each sample was used for western blot analysis with indicated antibodies. **b **analysis of EMT markers and TGF-β signaling pathways (both SMADs and non-SMADs) following treatment of BE2C cells with 30 and 60 µM of copper chelator of TM for 24 hours. **c **evaluating inhibition of TGF-β cleavage and activation in the cell lysate of BE2C cells treated with TM (0 & 60µM) for 24 hours. **Figure S5. a** The concentration of TGF-β in the TH-MYCN neuroblastoma mouse model sera treated with TEPA. TH-MYCN mice were treated with 400 mg/Kg TEPA for 7 days and the TGF-β expression level was analyzed in the sera of mice using a multiplex cytokine assay. Significance was determined by unpaired t-test with p-value=0.0007. **b** Analysis of the expression level of TGF-β in MDA-MB-231 and SH-SY5Y cells by Real-Time PCR. Briefly, the expression of TGF-β in the mentioned cells was analyzed 24 hours after treatment with specific amounts of TEPA. GUSB was used as an internal control. Significance was determined by unpaired t-test with p-value=0.0187 and p-value= 0.0060 for TNBC and NB, respectively. **c&d** Active and latent TGF-β in BE2C and MDA-MB-231 cell supernatants and DIPG007 & DIPG010 cell lysates. **Figure S6.** Heatmap highlighting TGF-β-related signalling pathways’ gene deregulation using RNA-seq data analysis for MDA-MB-231 cells treated with TEPA for 8 hours and 24 hours compared to the non-treated controls. RNA-seq data revealed that many target genes related to inhibiting EMT, immune evasion, and suppressing tumor growth downstream of TGF-β /SMAD, TGF-β/AKT/mTOR, TGF-β/RAS/RAF/MEK/ERK, and TGF-β/WNT/β-catenin signalling are dysregulated with TEPA. **Figure S7.** Confocal images (inverted for signal visibility) showing epithelial and mesenchymal cell state markers in BE2C and SH-SY5Y cells across control or TEPA treatment conditions. Violin plots showing quantification of single cell mean fluorescence intensities and quantification of single-cell nuclear-to cytoplasmic (N:C) mean intensity ratio. **Figure S8.** Immunohistochemistry of E-cadherin in 4T1.2 Mice model. CTRL is vehicle-treated control group and TEPA is mice treated with 800mg/kg TEPA). The E-cadherin immunohistochemistry was performed on Leica Bond RX (Leica) using BOND Polymer Refine Detection DAB kit (Leica). The slides were deparaffinized using BOND Dewax Solution (Leica) and epitope retrieval was performed using BOND Epitope retrieval solution 1 for 20min on the Leica BOND RX automated system. E-cadherin (24E10) (Cell Signaling, #3195) was diluted to 1:100 and incubated at room for 1 hour. Significance was determined by unpaired t-test with p-value=0.0438. **Table S1.** Gene signature of several important cancer-related signaling pathways in MDA-MB-231 TNBC cells treated with specific doses of TEPA for 8 and 24 hours. Genes with blue color are downregulated, and genes with red color are upregulated. **Table S2.** List and information of antibodies used for immunofluorescence staining. **Table S3.** List and information of antibodies used for western blot experiments.

## Data Availability

All data generated or analyzed during this study are included in this published article (and its Additional files).

## Abbreviations

DIPG: Diffuse intrinsic pontine glioma
DEGs: Differentially expressed genes
EMT: Epithelial-mesenchymal transition
GSEA: Gene set enrichment analysis
NB: Neuroblastoma
PI3K: Phosphatidylinositol 3- kinase
R-SMAD: Receptor-regulated SMAD
TNBC: Triple-negative breast cancer
TGF-β: Transforming growth factor -β
TEPA: Tetraethylenepentamine
VIM: Vimentin

## References

[1] Kalluri R, Weinberg RA (2009). The basics of epithelial-mesenchymal transition. J Clin Investig.

[2] Zeisberg M, Neilson EG (2009). Biomarkers for epithelial-mesenchymal transitions. J Clin Investig.

[3] Chaffer CL, Weinberg RA (2011). A perspective on cancer cell metastasis. Science.

[4] Valastyan S, Weinberg RA (2011). Tumor metastasis: molecular insights and evolving paradigms. Cell.

[5] Aiello NM, Kang Y (2019). Context-dependent EMT programs in cancer metastasis. J Exp Med.

[6] Liu X, Yun F, Shi L, Li Z-H, Luo N-R, Jia Y-F (2015). Roles of signaling pathways in the epithelial-mesenchymal transition in cancer. Asian Pac J Cancer Prev.

[7] Yaguchi T, Sumimoto H, Kudo-Saito C, Tsukamoto N, Ueda R, Iwata-Kajihara T (2011). The mechanisms of cancer immunoescape and development of overcoming strategies. Int J Hematol.

[8] Ribatti D, Tamma R, Annese T (2020). Epithelial-mesenchymal transition in cancer: a historical overview. Transl Oncol.

[9] Connolly EC, Freimuth J, Akhurst RJ (2012). Complexities of TGF-β targeted cancer therapy. Int J Biol Sci.

[10] Katz LH, Li Y, Chen J-S, Muñoz NM, Majumdar A, Chen J (2013). Targeting TGF-β signaling in cancer. Expert Opin Ther Targets.

[11] Xu J, Lamouille S, Derynck R (2009). TGF-β-induced epithelial to mesenchymal transition. Cell Res.

[12] Derynck R, Turley SJ, Akhurst RJ (2021). TGFβ biology in cancer progression and immunotherapy. Nat Rev Clin Oncol.

[13] Teixeira AF, Ten Dijke P, Zhu H-J (2020). On-target anti-TGF-β therapies are not succeeding in clinical cancer treatments: what are remaining challenges?. Front Cell Dev Biol.

[14] Bierie B, Moses HL (2010). Transforming growth factor beta (TGF-β) and inflammation in cancer. Cytokine Growth Factor Rev.

[15] Gómez-Gil V (2021). Therapeutic implications of TGFβ in cancer treatment: a systematic review. Cancers.

[16] Dickson MC, Martin JS, Cousins FM, Kulkarni AB, Karlsson S, Akhurst RJ (1995). Defective haematopoiesis and vasculogenesis in transforming growth factor-beta 1 knock out mice. Development.

[17] Anderton MJ, Mellor HR, Bell A, Sadler C, Pass M, Powell S (2011). Induction of heart valve lesions by small-molecule ALK5 inhibitors. Toxicol Pathol.

[18] Wiercinska E, Naber HP, Pardali E. The TGF-/Smad pathway induces breast cancer cell invasion through the up-regulation of matrix metalloproteinase 2 and 9 in a spheroid invasion model system. 10.1007/s10549-010-1147-x 20821046

[19] Stuelten CH, Byfield SD, Arany PR, Karpova TS, Stetler-Stevenson WG, Roberts AB (2005). Breast cancer cells induce stromal fibroblasts to express MMP-9 via secretion of TNF-α and TGF-β. J Cell Sci.

[20] Ashraf ST, Obaid A, Saeed MT, Naz A, Shahid F, Ahmad J (2019). Formal model of the interplay between TGF-β1 and MMP-9 and their dynamics in hepatocellular carcinoma. Math Biosci Eng.

[21] Yamahana H, Terashima M, Takatsuka R, Asada C, Suzuki T, Uto Y (2021). TGF-β1 facilitates MT1-MMP-mediated proMMP-9 activation and invasion in oral squamous cell carcinoma cells. Biochem Biophys Rep.

[22] Seomun Y, Kim JT, Joo CK (2008). MMP-14 mediated MMP-9 expression is involved in TGF-beta1-induced keratinocyte migration. J Cell Biochem.

[23] Augoff K, Hryniewicz-Jankowska A, Tabola R, Stach K (2022). MMP9: a tough target for targeted therapy for cancer. Cancers.

[24] Dong H, Diao H, Zhao Y, Xu H, Pei S, Gao J (2019). Overexpression of matrix metalloproteinase-9 in breast cancer cell lines remarkably increases the cell malignancy largely via activation of transforming growth factor beta/SMAD signalling. Cell Prolif.

[25] Hochheuser C, Windt LJ, Kunze NY, de Vos DL, Tytgat GA, Voermans C (2021). Mesenchymal stromal cells in neuroblastoma: exploring crosstalk and therapeutic implications. Stem Cells Dev.

[26] Bierie B, Moses HL (2006). TGFβ: the molecular Jekyll and Hyde of cancer. Nat Rev Cancer.

[27] Bierie B, Stover DG, Abel TW, Chytil A, Gorska AE, Aakre M (2008). Transforming growth factor–β regulates mammary carcinoma cell survival and interaction with the adjacent microenvironment. Can Res.

[28] Bhola NE, Balko JM, Dugger TC, Kuba MG, Sánchez V, Sanders M (2013). TGF-β inhibition enhances chemotherapy action against triple-negative breast cancer. J Clin Investig.

[29] Blockhuys S, Celauro E, Hildesjö C, Feizi A, Stål O, Fierro-González J (2017). Defining the human copper proteome and analysis of its expression variation in cancers. Metallomics.

[30] Liu YL, Bager CL, Willumsen N, Ramchandani D, Kornhauser N, Ling L (2021). Tetrathiomolybdate (TM)-associated copper depletion influences collagen remodeling and immune response in the pre-metastatic niche of breast cancer. NPJ Breast Cancer.

[31] Lelièvre P, Sancey L, Coll J-L, Deniaud A, Busser B (2020). The multifaceted roles of copper in cancer: a trace metal element with dysregulated metabolism, but also a target or a bullet for therapy. Cancers.

[32] Michniewicz F, Saletta F, Rouaen JR, Hewavisenti RV, Mercatelli D, Cirillo G (2021). Copper: an intracellular achilles’ heel allowing the targeting of epigenetics, kinase pathways, and cell metabolism in cancer therapeutics. Chem Med Chem.

[33] Schmidt K, Ralle M, Schaffer T, Jayakanthan S, Bari B, Muchenditsi A (2018). ATP7A and ATP7B copper transporters have distinct functions in the regulation of neuronal dopamine-β-hydroxylase. J Biol Chem.

[34] Zecca L, Stroppolo A, Gatti A, Tampellini D, Toscani M, Gallorini M (2004). The role of iron and copper molecules in the neuronal vulnerability of locus coeruleus and substantia nigra during aging. Proc Natl Acad Sci.

[35] Xiao T, Ackerman CM, Carroll EC, Jia S, Hoagland A, Chan J (2018). Copper regulates rest-activity cycles through the locus coeruleus-norepinephrine system. Nat Chem Biol.

[36] Panichelli P, Villano C, Cistaro A, Bruno A, Barbato F, Piccardo A (2016). Imaging of brain tumors with copper-64 chloride: early experience and results. Cancer Biother Radiopharm.

[37] Brewer GJ, Askari F, Dick RB, Sitterly J, Fink JK, Carlson M (2009). Treatment of Wilson's disease with tetrathiomolybdate: V. Control of free copper by tetrathiomolybdate and a comparison with trientine. Transl Res.

[38] Pan Q, Kleer CG, Van Golen KL, Irani J, Bottema KM, Bias C (2002). Copper deficiency induced by tetrathiomolybdate suppresses tumor growth and angiogenesis. Can Res.

[39] Pan Q, Rosenthal DT, Bao L, Kleer CG, Merajver SD (2009). Antiangiogenic tetrathiomolybdate protects against Her2/neu-induced breast carcinoma by hypoplastic remodeling of the mammary gland. Clin Cancer Res.

[40] Valli E, Lerra L, Kimpton K, Saletta F, Giorgi FM, Mercatelli D (2020). Intratumoral copper modulates PD-L1 expression and influences tumor immune evasion. Can Res.

[41] Han Y, Liu D, Li L (2020). PD-1/PD-L1 pathway: current researches in cancer. Am J Cancer Res.

[42] Kim S-H, Redvers RP, Chi LH, Ling X, Lucke AJ, Reid RC (2018). Identification of brain metastasis genes and therapeutic evaluation of histone deacetylase inhibitors in a clinically relevant model of breast cancer brain metastasis. Dis Models Mech.

[43] Joseph MJ, Dangi-Garimella S, Shields MA, Diamond ME, Sun L, Koblinski JE (2009). Slug is a downstream mediator of transforming growth factor-β1-induced matrix metalloproteinase-9 expression and invasion of oral cancer cells. J Cell Biochem.

[44] Ferrari-Amorotti G, Chiodoni C, Shen F, Cattelani S, Soliera AR, Manzotti G (2014). Suppression of invasion and metastasis of triple-negative breast cancer lines by pharmacological or genetic inhibition of slug activity. Neoplasia.

[45] Voli F, Valli E, Lerra L, Kimpton K, Saletta F, Giorgi FM (2020). Intratumoral copper modulates PD-L1 expression and influences tumor immune evasion. Can Res.

[46] Sammons S, Brady D, Vahdat L, Salama AK (2016). Copper suppression as cancer therapy: The rationale for copper chelating agents in BRAF V600 mutated melanoma. Melanoma Manag.

[47] Baldari S, Di Rocco G, Heffern MC, Su TA, Chang CJ, Toietta G (2019). Effects of copper chelation on BRAFV600E positive colon carcinoma cells. Cancers.

[48] Georgakopoulos-Soares I, Chartoumpekis DV, Kyriazopoulou V, Zaravinos A (2020). EMT factors and metabolic pathways in cancer. Front Oncol.

[49] Gupta PB, Pastushenko I, Skibinski A, Blanpain C, Kuperwasser C (2019). Phenotypic plasticity: driver of cancer initiation, progression, and therapy resistance. Cell Stem Cell.

[50] Mu Y, Gudey SK, Landström M (2012). Non-smad signaling pathways. Cell Tissue Res.

[51] Luo K (2017). Signaling cross talk between TGF-β/Smad and other signaling pathways. Cold Spring Harb Perspect Biol.

[52] Zhang YE (2017). Non-Smad signaling pathways of the TGF-β family. Cold Spring Harb Perspect Biol.

[53] Miyazono K, Katsuno Y, Koinuma D, Ehata S, Morikawa M (2018). Intracellular and extracellular TGF-β signaling in cancer: some recent topics. Front Med.

[54] Lv Z-D, Kong B, Li J-G, Qu H-L, Wang X-G, Cao W-H (2013). Transforming growth factor-β 1 enhances the invasiveness of breast cancer cells by inducing a Smad2-dependent epithelial-to-mesenchymal transition. Oncol Rep.

[55] Shao J-B, Gao Z-M, Huang W-Y, Lu Z-B (2017). The mechanism of epithelial-mesenchymal transition induced by TGF-β1 in neuroblastoma cells. Int J Oncol.

[56] Whiteside EJ, Jackson MM, Herington AC, Edwards DR, Harvey MB (2001). Matrix metalloproteinase-9 and tissue inhibitor of metalloproteinase-3 are key regulators of extracellular matrix degradation by mouse embryos. Biol Reprod.

[57] Pietruszewska W, Bojanowska-Poźniak K, Kobos J (2016). Matrix metalloproteinases MMP1, MMP2, MMP9 and their tissue inhibitors TIMP1, TIMP2, TIMP3 in head and neck cancer: an immunohistochemical study. Otolaryngol Pol.

[58] Chen G, Ge D, Zhu B, Shi H, Ma Q (2020). Upregulation of matrix metalloproteinase 9 (MMP9)/tissue inhibitor of metalloproteinase 1 (TIMP1) and MMP2/TIMP2 ratios may be involved in lipopolysaccharide-induced acute lung injury. J Int Med Res.

[59] Chakravarthy A, Khan L, Bensler NP, Bose P, De Carvalho DD (2018). TGF-β-associated extracellular matrix genes link cancer-associated fibroblasts to immune evasion and immunotherapy failure. Nat Commun.

[60] Alsuliman A, Colak D, Al-Harazi O, Fitwi H, Tulbah A, Al-Tweigeri T (2015). Bidirectional crosstalk between PD-L1 expression and epithelial to mesenchymal transition: significance in claudin-low breast cancer cells. Mol Cancer.

[61] Kalimutho M, Sinha D, Mittal D, Srihari S, Nanayakkara D, Shafique S (2019). Blockade of PDGFRβ circumvents resistance to MEK-JAK inhibition via intratumoral CD8+ T-cells infiltration in triple-negative breast cancer. J Exp Clin Cancer Res.

[62] Khan A, Gamble LD, Upton DH, Ung C, Yu DM, Ehteda A (2021). Dual targeting of polyamine synthesis and uptake in diffuse intrinsic pontine gliomas. Nat Commun.

[63] Van der Maaten L, Hinton G. Visualizing data using t-SNE. Journal of machine learning research. 2008;9(11).

[64] Wickham H, Wickham H (2016). Data analysis. ggplot2.

[65] Bioinformatics B. https://www.bioinformatics.babraham.ac.uk/projects/fastqc/.

[66] Kim D, Langmead B, Salzberg SL (2015). HISAT: a fast spliced aligner with low memory requirements. Nat Methods.

[67] Liao Y, Smyth GK, Shi W (2014). featureCounts: an efficient general purpose program for assigning sequence reads to genomic features. Bioinformatics.

[68] Ensembl. 2022 https://pubmed.ncbi.nlm.nih.gov/34791404/.

[69] Love MI, Huber W, Anders S (2014). Moderated estimation of fold change and dispersion for RNA-seq data with DESeq2. Genome Biol.

[70] Giorgi FM, Ceraolo C, Mercatelli D (2022). The R language: an engine for bioinformatics and data science. Life.

[71] Kuleshov MV, Jones MR, Rouillard AD, Fernandez NF, Duan Q, Wang Z (2016). Enrichr: a comprehensive gene set enrichment analysis web server 2016 update. Nucleic Acids Res.

[72] Korotkevich G, Sukhov V, Sergushichev A. Fast gene set enrichment analysis. bioRxiv. 2019:060012.

[73] Korotkevich G, Sukhov V, Budin N, Shpak B, Artyomov MN, Sergushichev A. Fast gene set enrichment analysis. bioRxiv. 2021:060012.

[74] Dolgalev I. MSigDB Gene Sets for Multiple Organisms in a Tidy Data Format. R package version 7.4.1 2021 https://cran.r-project.org/package=msigdbr.

[75] Mercatelli D, Lopez-Garcia G, Giorgi FM (2020). corto: a lightweight R package for gene network inference and master regulator analysis. Bioinformatics.

[76] Giorgi FM, Del Fabbro C, Licausi F (2013). Comparative study of RNA-seq-and microarray-derived coexpression networks in Arabidopsis thaliana. Bioinformatics.

