# Supplementary material for: Copper chelation suppresses epithelial-mesenchymal transition by inhibition of canonical and non-canonical TGF-β signaling pathways in cancer
Source: Cell Biosci. 2023 Jul 21;13:132. doi: 10.1186/s13578-023-01083-7 (PMC10362738; doi:10.1186/s13578-023-01083-7)
Supplement: Supplementary file 1 — Additional file1: Figure S1. Analysis of cell migration following treating cells with TEPA. a & b Cell migration analysis by scratch wound assay for MDA-MB-231 and SH-SY5Y cells, respectively. Pink areas indicate the scratch-wound area. This experiment have done as triplicate and significance was confirmed by p-value <0.001. Figure S2. Gene expression changes in TEPA-treated MDA-MB-231 cells. Volcano plots showing gene expression changes after 8 (a) and 24 (b) hours of TEPA treatment. The top significant 25 up and top 25 down-regulated genes are labeled. c K-means clustering classifies gene expression changes into 4 clusters. Genes included in cluster 4 show a time-dependent downregulation. d Top 10 MSigDB Hallmark enriched gene sets. Significant enrichments of MSigDB gene sets were evaluated through enrichment analysis performed with EnrichR. Color intensity is referred to the enrichment score computed by EnrichR and calculated as follows: combined score = log(p) * z, where p is the Fisher exact test p-value, and z is the z-score for deviation from expected rank. Asterisks mean a corrected p-value for multiple testing < 0.05. Figure S3. Master Regulator Analysis. SNAI2 sub-network downregulation in TEPA-treated cells at 8 (up) and 24 hours (down). The top 12 highest-likelihood targets are shown on the right side. The genes in each network are shown in a barcode-like diagram showing all transcriptome genes by means of their differential expression upon TEPA treatment, from the most downregulated (left) to the most upregulated(right). A blue background on the NES box is used to indicate a negative enrichment (or repression of the corresponding co-expression network). Figure S4. a Western blot analysis of MMP-2 in cell supernatant, and mTOR, phospho-mTOR (Ser2448), and E-cadherin (CDH1) in cell lysate. For MMP-2, cells were treated with specific amount of TEPA in serum-free media for 24 hours. Then cell supernatants were collected, and soluble proteins were concentrat [file 13578_2023_1083_MOESM1_ESM.docx]

Additional files for

**Copper chelation suppresses epithelial-mesenchymal transition by inhibition of canonical and non-canonical TGF-β Signaling pathways in cancer**

**Authors: Ensieh. M. Poursani^1, 2^*, Daniele Mercatelli^3^*, Prahlad Raninga^4^*, Jessica L. Bell^1,2^, Federica Saletta^1,2^, Felix V. Kohane^2,10^, Daniel P. Neumann^2,10^, Ye Zheng^2^, Jourdin Rouaen^1,2^, Toni R. Jue^1,2^, Filip T. Michniewicz^1,2^, Piper Schadel^1,2^, Erin Kasiou^1,2^, Maria Tsoli^1,2^, Giuseppe Cirillo^5^, Shafagh Waters^2^, Tyler Shai-Hee^1,2^, Riccardo Cazzoli^6^, Merryn Brettle^7^, Iveta Slapetova^7^, Maria Kasherman^7^,** **Renee Whan^7^, Fernando Souza-Fonseca-Guimaraes^11^, Linda Vahdat^8^, David Ziegler^1,2,9^, John G. Lock^2^, Federico M. Giorgi^3^, Kum Kum Khanna^4^* & Orazio Vittorio^1,2^*.**

***Equally contributed**

**Corresponding Author: O Vittorio, Email: ovittorio@ccia.org.au**

**Affiliations**

1. Children’s Cancer Institute Australia, Lowy Cancer Research Centre, UNSW Australia, Sydney, Australia.
2. School of Biomedical Sciences, Faculty of Medicine and Health, UNSW Sydney, NSW, Australia
3. Department of Pharmacy and Biotechnology, University of Bologna, Bologna Italy.
4. QIMR Berghofer Medical Research Institute, Brisbane, QLD, Australia
5. Department of Pharmacy, Health and Nutritional Sciences, University of Calabria, Rende, Italy
6. Department of Experimental Oncology, IEO, European Institute of Oncology IRCCS, Milan, Italy
7. Katharina Gauss Light Microscopy Facility, UNSW Sydney, NSW, Australia
8. Dartmouth Cancer Center, Lebanon, New Hampshire, USA
9. Kids Cancer Centre, Sydney Children’s Hospital, Randwick, NSW, Australia
10. Garvan Institute of Medical Research, Darlinghurst, NSW, Australia

11. The University of Queensland Diamantina Institute, The University of Queensland, Woolloongabba, QLD, Australia

**This file includes:**

Figures. S1 to S8

Tables S1 to S3

Supplementary Figures

Figure S1.


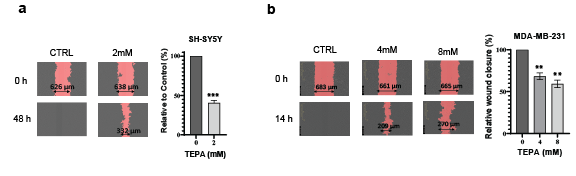


**Figure S1**- Analysis of cell migration following treating cells with TEPA. **a & b** Cell migration analysis by scratch wound assay for MDA-MB-231 and SH-SY5Y cells, respectively. Pink areas indicate the scratch-wound area. This experiment have done as triplicate and significance was confirmed by p-value <0.001.

**Figure S2.**


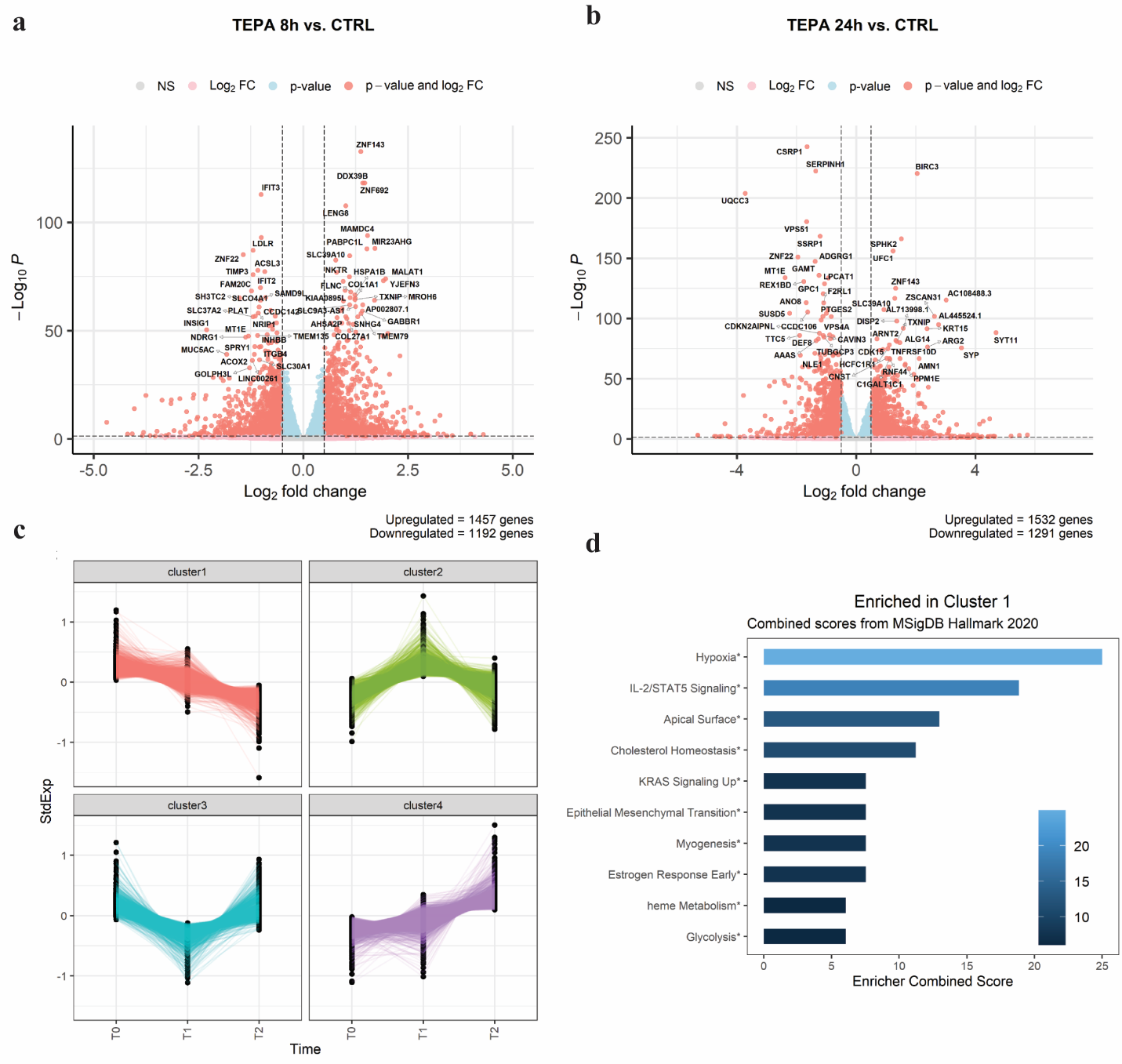


**Figure S2-** Gene expression changes in TEPA-treated MDA-MB-231 cells. Volcano plots showing gene expression changes after 8 (**a**) and 24 (**b**) hours of TEPA treatment. The top significant 25 up and top 25 down-regulated genes are labeled. **c** K-means clustering classifies gene expression changes into 4 clusters. Genes included in cluster 4 show a time-dependent downregulation. **d** Top 10 MSigDB Hallmark enriched gene sets. Significant enrichments of MSigDB gene sets were evaluated through enrichment analysis performed with EnrichR. Color intensity is referred to the enrichment score computed by EnrichR and calculated as follows: combined score = log(p) * z, where p is the Fisher exact test p-value, and z is the z-score for deviation from expected rank. Asterisks mean a corrected p-value for multiple testing < 0.05.

**Figure S3.**


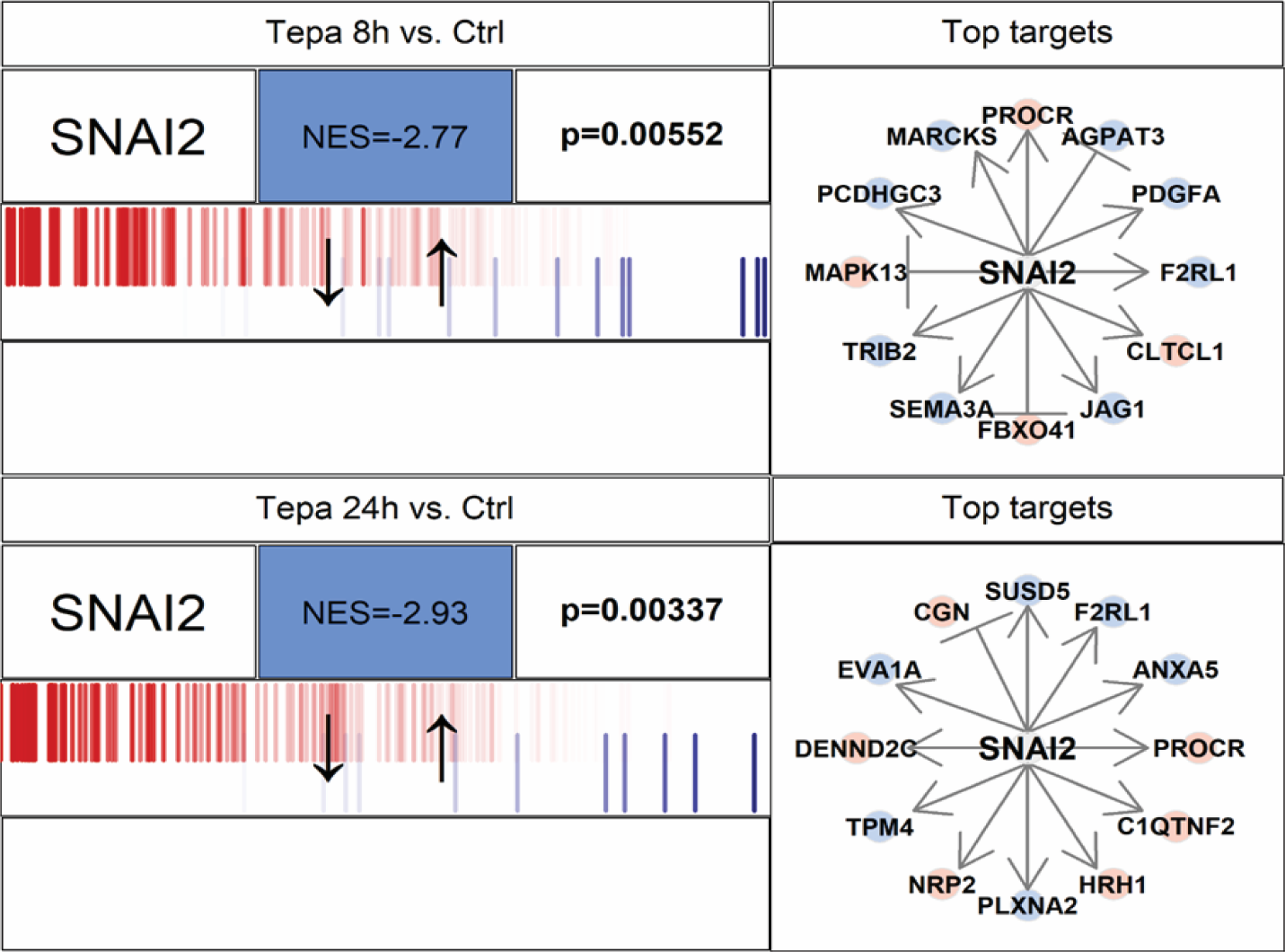


**Figure S3- Master Regulator Analysis.** SNAI2 sub-network downregulation in TEPA-treated cells at 8 (up) and 24 hours (down). The top 12 highest-likelihood targets are shown on the right side. The genes in each network are shown in a barcode-like diagram showing all transcriptome genes by means of their differential expression upon TEPA treatment, from the most downregulated (left) to the most upregulated(right). A blue background on the NES box is used to indicate a negative enrichment (or repression of the corresponding co-expression network).

**Figure S4.**


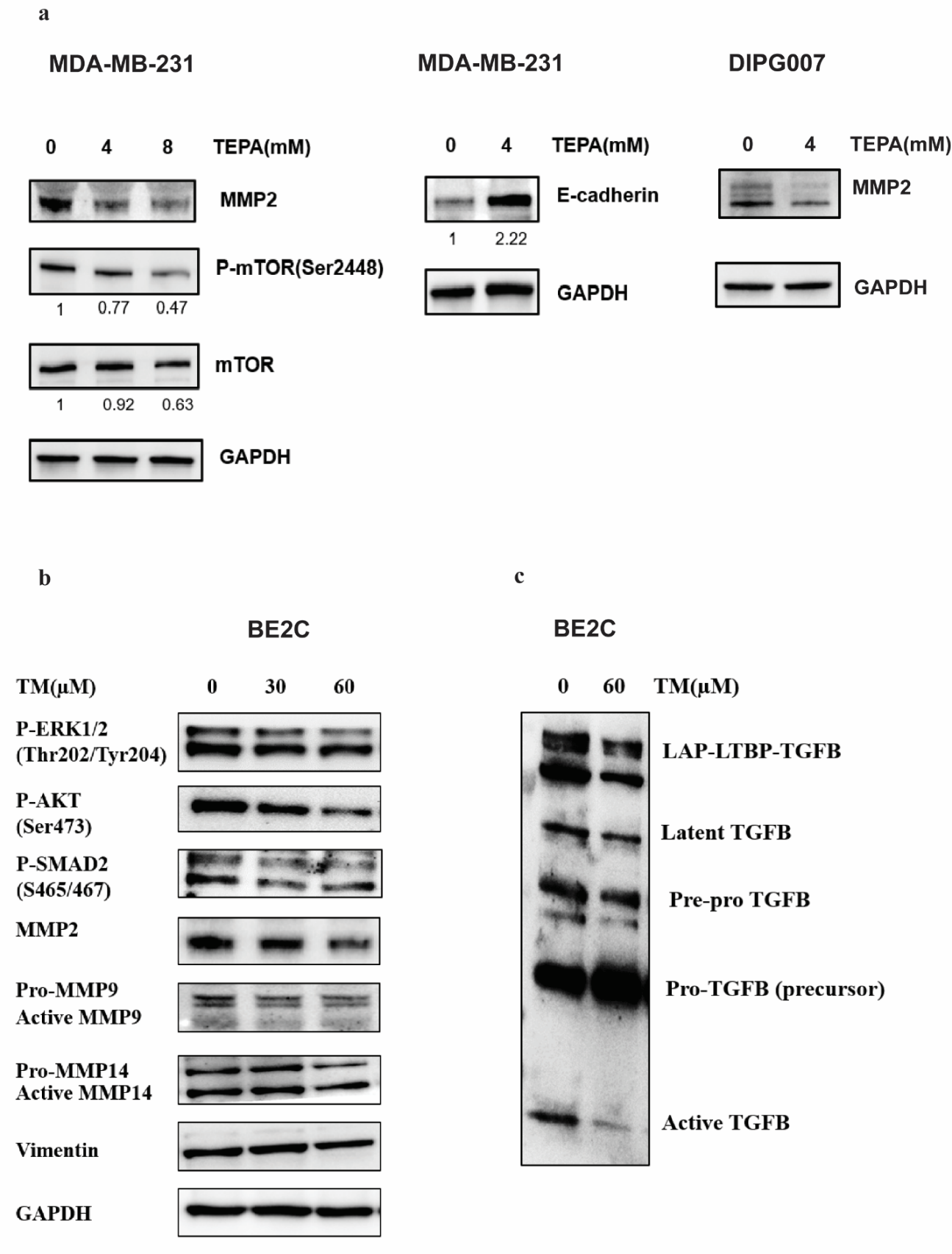


# Figure S4- a Western blot analysis of MMP2 in cell supernatant, and mTOR, phospho-mTOR (Ser2448), and E-cadherin (CDH1) in cell lysate. For MMP2, cells were treated with specific amount of TEPA in serum-free media for 24 hours. Then cell supernatants were collected, and soluble proteins were concentrated using Ultracel-10 regenerated cellulose membrane (Amicon® Ultra-15 Centrifugal Filter Unit, Merck). 20 µg of each sample was used for western blot analysis with indicated antibodies. b analysis of EMT markers and TGF-β signaling pathways (both SMADs and non-SMADs) following treatment of BE2C cells with 30 and 60 µM of copper chelator of TM for 24 hours. c evaluating inhibition of TGF-β cleavage and activation in the cell lysate of BE2C cells treated with TM (0 & 60µM) for 24 hours.

**Figure S5.**


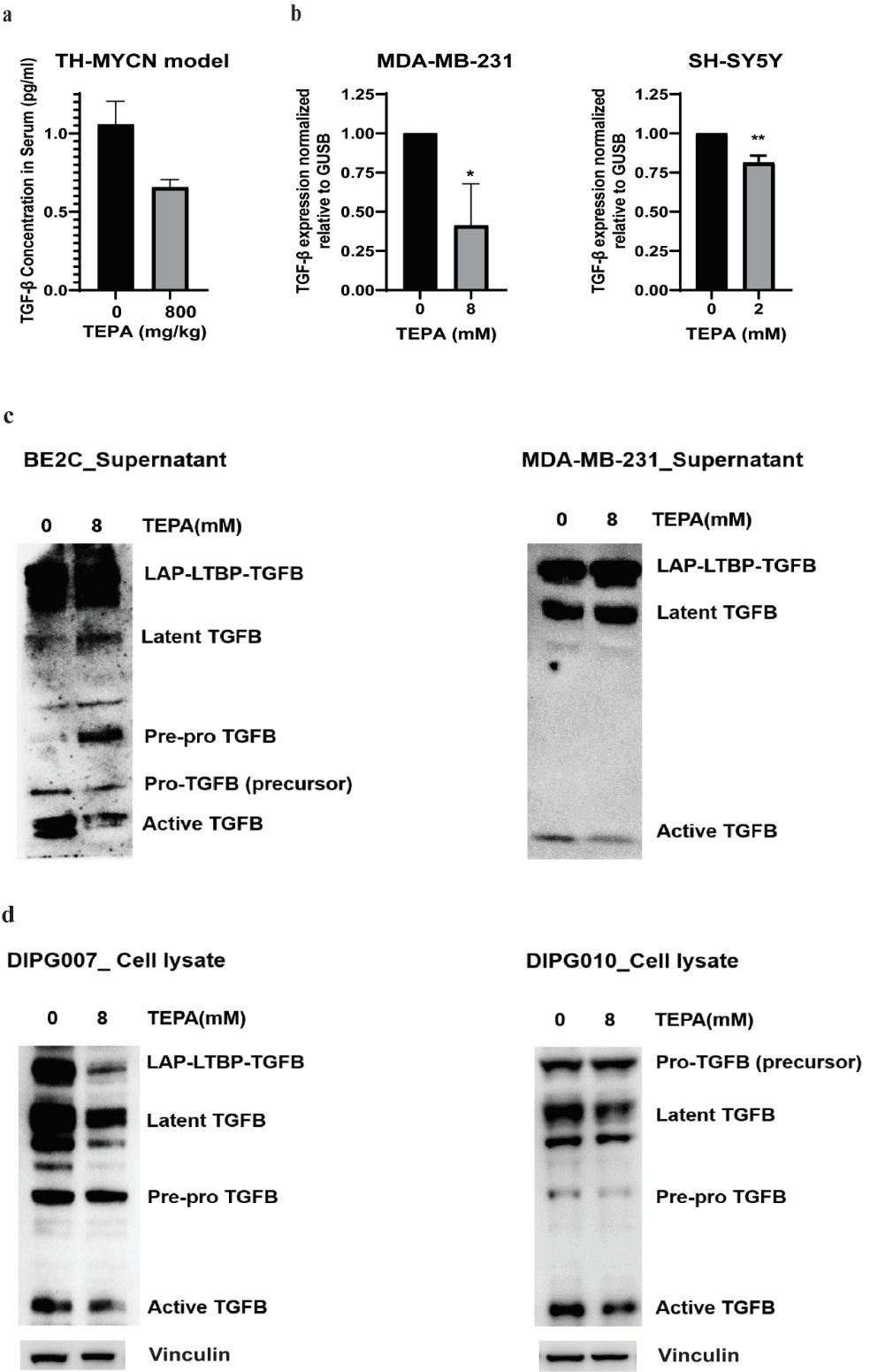


**Figure S5. a** The concentration of TGF-β in the TH-MYCN neuroblastoma mouse model sera treated with TEPA. TH-MYCN mice were treated with 400 mg/Kg TEPA for 7 days and the TGF-β expression level was analyzed in the sera of mice using a multiplex cytokine assay. Significance was determined by unpaired t-test with p-value=0.0007. **b** Analysis of the expression level of TGF-β in MDA-MB-231 and SH-SY5Y cells by Real-Time PCR. Briefly, the expression of TGF-β in the mentioned cells was analyzed 24 hours after treatment with specific amounts of TEPA. GUSB was used as an internal control. Significance was determined by unpaired t-test with p-value=0.0187 and p-value= 0.0060 for TNBC and NB, respectively. **c&d** Active and latent TGF-β in BE2C and MDA-MB-231 cell supernatants and DIPG007 & DIPG010 cell lysates.

**Figure S6.**
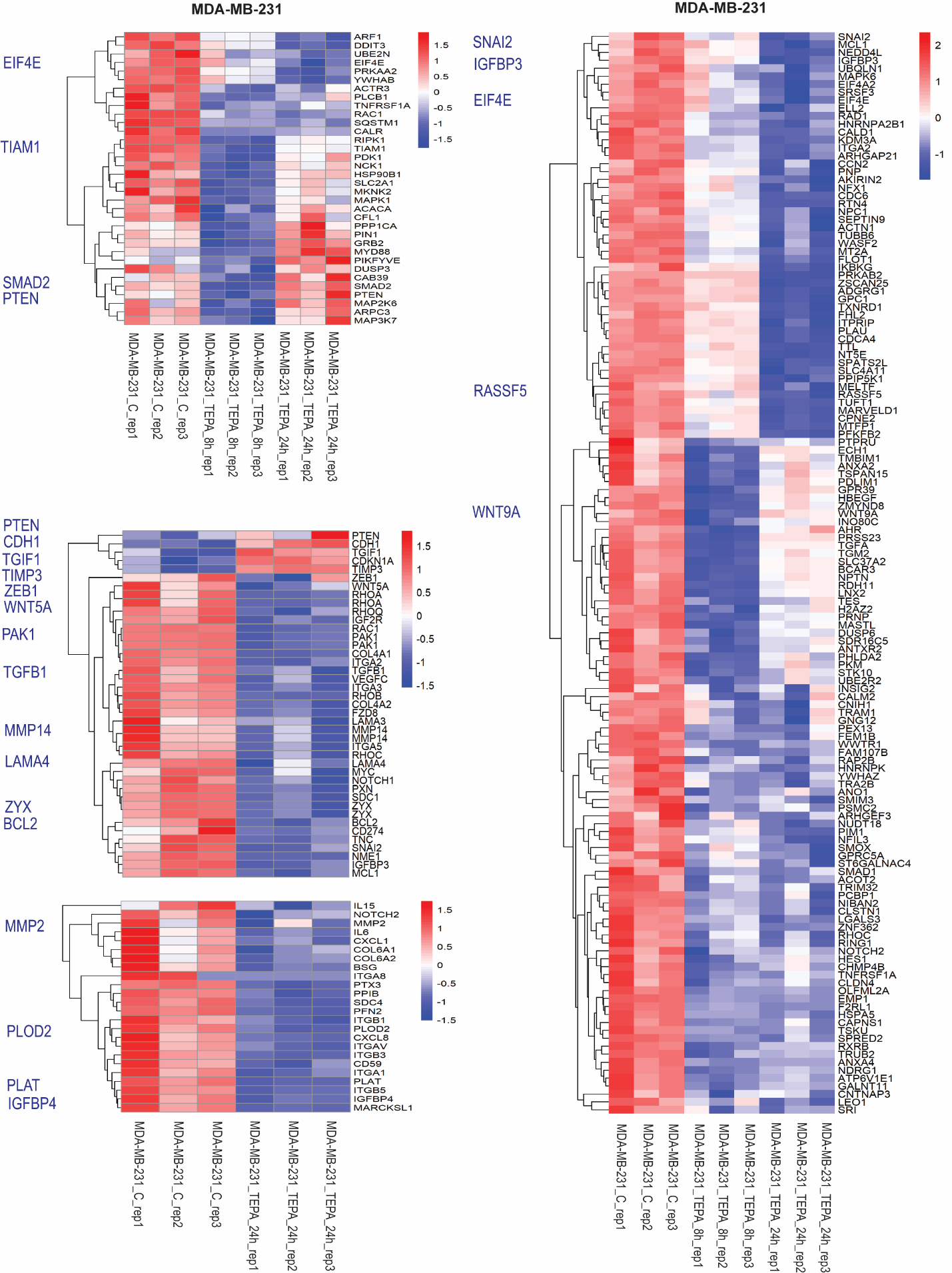


**Figure S6.** Heatmap highlighting TGF-β-related signalling pathways’ gene deregulation using RNA-seq data analysis for MDA-MB-231 cells treated with TEPA for 8 hours and 24 hours compared to the non-treated controls. RNA-seq data revealed that many target genes related to inhibiting EMT, immune evasion, and suppressing tumor growth downstream of TGF-β /SMAD, TGF-β/AKT/mTOR, TGF-β/RAS/RAF/MEK/ERK, and TGF-β/WNT/β-catenin signalling are dysregulated with TEPA.

**Figure S7.**


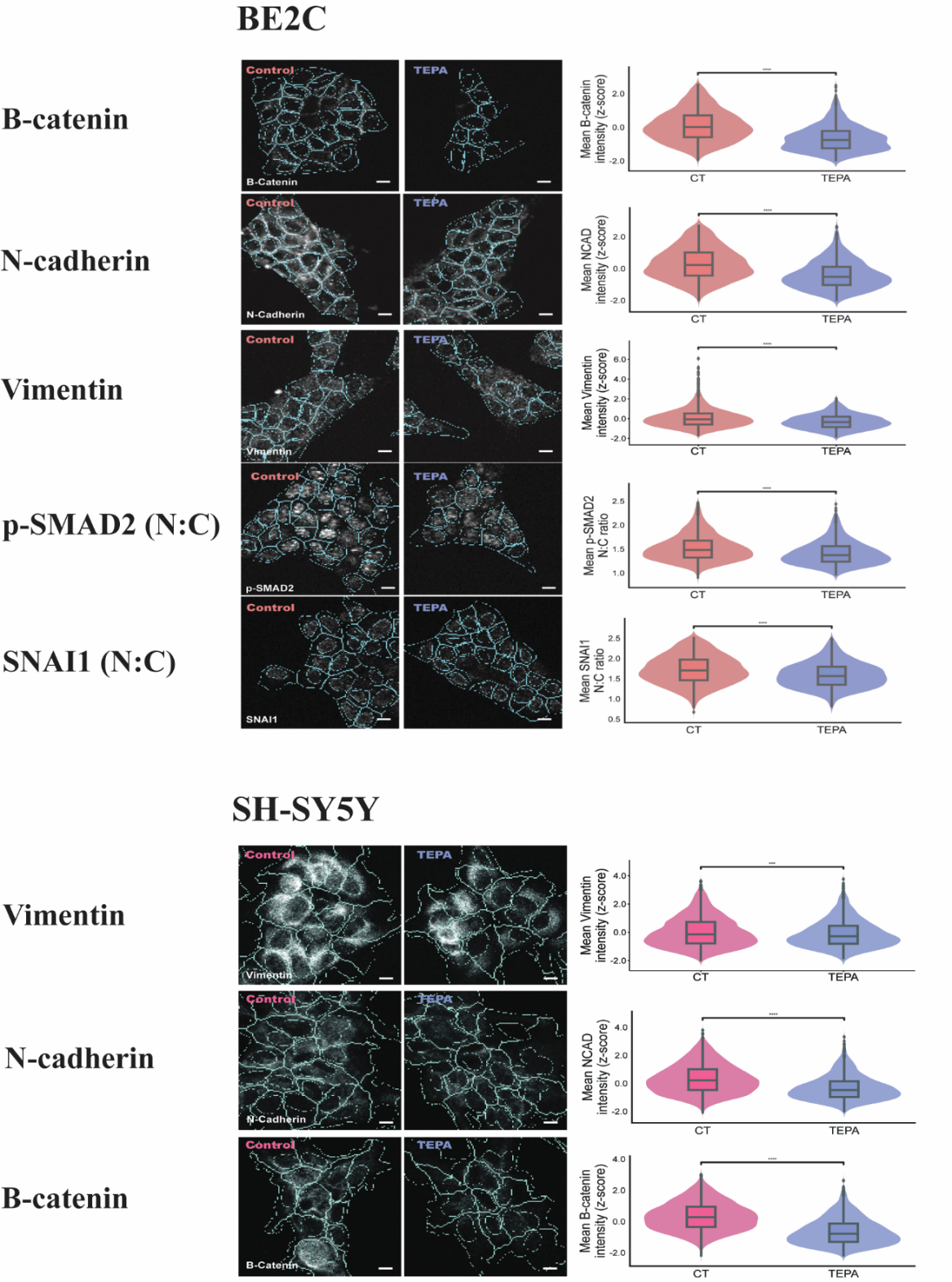


**Figure S7**. Confocal images (inverted for signal visibility) showing epithelial and mesenchymal cell state markers in BE2C and SH-SY5Y cells across control or TEPA treatment conditions. Violin plots showing quantification of single cell mean fluorescence intensities and quantification of single-cell nuclear-to cytoplasmic (N:C) mean intensity ratio.

**Figure S8.**


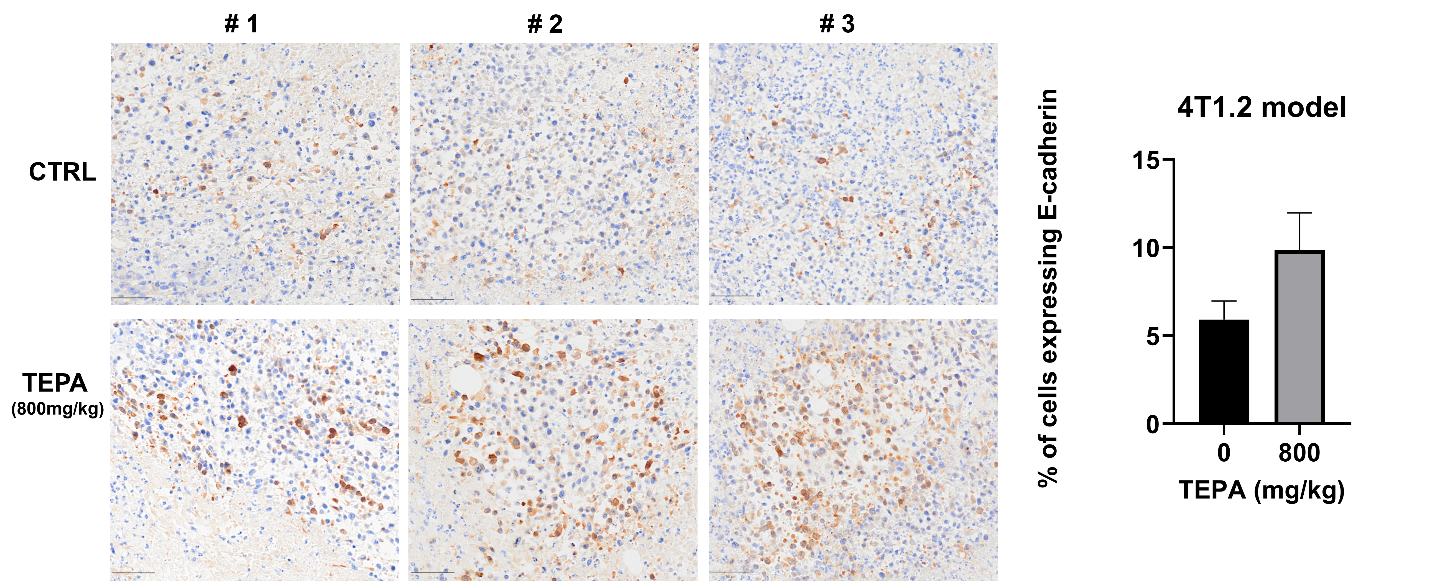


**Figure S8.** Immunohistochemistry of E-cadherin in 4T1.2 Mice model. CTRL is vehicle-treated control group and TEPA is mice treated with 800mg/kg TEPA). The E-cadherin immunohistochemistry was performed on Leica Bond RX (Leica) using BOND Polymer Refine Detection DAB kit (Leica). The slides were deparaffinized using BOND Dewax Solution (Leica) and epitope retrieval was performed using BOND Epitope retrieval solution 1 for 20min on the Leica BOND RX automated system. E-cadherin (24E10) (Cell Signaling, #3195) was diluted to 1:100 and incubated at room for 1 hour. Significance was determined by unpaired t-test with p-value=0.0438.

Table S1.

| Signaling pathways (KEGG) | | Genes Symbol | |
| --- | --- | --- | --- |
|  | 8 hours | | 24 hours |
| TGF-β/SMAD2/3 Signaling | | TGFBR2, SPARC | SNAI2, RAC2, BCL2, CDKN1A |
| PI3K/AKT Signaling | | PIK3C2B, PIK3IP1, HSP90 |  |
| mTOR Signaling | | TNFRSF1A | WNT5A, eIF4E |
| MAPK Signaling | | TGF-α, MKNK2, DUSP6, TNFRSF1A | RAC2 |
| Ras/Raf/Erk Signaling | | TIAM1 | RASSF5, IKBKG |
| TLR/NFκB Signaling | | TLR2, TLR3, TRAF6, RAC2, MAP2K6, RIPK1 | IKBKG, PLAU |
| IGF Signaling | | IGFBP-1, IGFBP-4, IGFBP-5, IGFBP-6, IGFBP7 | IGFBP-3, ITGA2, ITGA3 |
| ECM receptor Signaling | | ITGA1, ITGAV, ITGB3, ITGB5 | COL4A1 |
| Focal adhesion | |  | PXN, LAMA4, ZYX |
| Inflammation & immune system suppression | | PTX3, CXCL1, CXCL8, IL15, IL6 | IL6R |
| Cell mobility, invasion & metastasis | | PLOD2, PLAT, MARCKSL1, CCR7, MMP11, MMP15 | PAK1, ZYX, TIMP3, TIMP4, CDH1 |
| Apoptosis & cell cycle inhibition | | BCL2L1 | BCL2, CDKN1A, CDKN2D |
| WNT Signaling | | NOTCH3, WNT9A, FZD1, FZD8, AXIN2, PFN2 | NOTCH1, WNT5A, WNT7B, WNT10B |
| Angiogenesis | | VEGFB |  |

**Table S1**- Gene signature of several important cancer-related signaling pathways in MDA-MB-231 TNBC cells treated with specific doses of TEPA for 8 and 24 hours. Genes with blue color are downregulated, and genes with red color are upregulated.

**Table S2.**

| **Antibody** | **Dilution** | **Cat number** | **Company** |
| --- | --- | --- | --- |
| **SNAI1** | 1:500 | 13099-1-AP | Proteintech |
| **phospho-Smad2(Ser465/467)** | 1:400 | 138D4 | Cell Signaling Technology |
| **β-catenin** | 1:500 | 71-2700 | ThermoFisher Scientific |
| **phospho-AKT(S473)** | 1:500 | 3787 | Cell Signaling Technology |
| **ZEB1** | 1:200 | 3396 | Cell Signalling Technology |
| **N-cadherin** | 1:100 | 14215S | Cell Signalling Technology |
| **Vimentin** | 1:500 | ab8978 | Abcam |
| **Anti-mouse AlexaFluor 488** | 1:1000 | 4408 | Cell Signalling Technology |
| **anti-rabbit AlexaFluor 555** | 1:1000 | 4413 | Cell Signalling Technology |
| **DAPI** | 1:2000 | D9542 | Sigma-Aldrich |
| **Phalloidin-Atto 647N** | 1:2000 | AD647N | ATTO-TEC |

**Table S2.** List and information of antibodies used for immunofluorescence staining.

**Table S3.**

| **Antibody** | **Cat number** | **Company** |
| --- | --- | --- |
| **Smad2** | ab40855 | Abcam |
| **phospho-Smad2(Ser465/467)** | 138D4 | Cell Signaling Technology |
| **AKT (pan)** | 11E7 | Cell Signaling Technology |
| **phospho-AKT(S473)** | 193H12 | Cell Signaling Technology |
| **Phospho-p44/42 MAPK(pERK1/2) (T202/Y204)** | D13.14.4E | Cell Signaling Technology |
| **p44/42 MAPK (ERK1/2)** | 9102S | Cell Signaling Technology |
| **MMP-9** | D6O3H | Cell Signaling Technology |
| **MMP-2** | A6247 | ABclonal |
| **MMP-14** | A2549 | ABclonal |
| **mTOR** | 2972 | Cell Signaling Technology |
| **Phospho-mTOR(Ser2448)** | 2971 | Cell Signaling Technology |
| **E-cadherin** | 24E10 | Cell Signaling Technology |
| **Vimentin** | D21H3 | Cell Signaling Technology |
| **GAPDH** | sc-365062 | Santa Cruz |
| **polyclonal HRP-conjugated goat anti-rabbit** | Dako | Denmark |
| **anti-mouse IgG secondary antibodies** | Glostrup | Denmark |

**Table S3.** List and information of antibodies used for western blot experiments.
